# Correlation of toxicities and efficacies of pemetrexed with clinical factors and single-nucleotide polymorphisms: a prospective observational study

**DOI:** 10.1186/s12885-023-11257-8

**Published:** 2023-08-26

**Authors:** Yuichiro Takeda, Go Naka, Yuki Katsuya, Konomi Kobayashi, Manabu Suzuki, Masao Hashimoto, Satoshi Hirano, Yukari Uemura

**Affiliations:** 1 Department of Clinical Laboratory, National Center for Global Health and Medicine, 1-21-1 Toyama Shinjuku-Ku, Tokyo, 162-8655 Japan; 2 Department of Respiratory Medicine, National Center for Global Health and Medicine, 1-21-1 Toyama Shinjuku-Ku, Tokyo, 162-8655 Japan; 3 Present Address: Department of Experimental Therapeutics, National Cancer Center Hospital, Tokyo, Japan; 4 Present Address: Division of Respiratory Medicine and Allergology, Department of Medicine, Teikyo University School of Medicine, Tokyo, Japan; 5 Department of Medical Oncology, Funabashi Municipal Medical Center, 1-21-1 Kanasugi, Funabashi, Chiba 273-8588 Japan; 6 Section of Biostatistics, Department of Clinical Research Center, The National Center for Global Health and Medicine Center, 1-21-1 Toyama Shinjuku-Ku, Tokyo, 162-8655 Japan

**Keywords:** Pemetrexed, Toxicities and efficacies, Single-nucleotide polymorphism, Betaine-homocysteine methyltransferase, Methionine cycle

## Abstract

**Background:**

Pemetrexed is an efficacious multi-targeted antifolate with acceptable toxicity for non-squamous non-small cell lung cancer (non-Sq NSCLC) and malignant pleural mesothelioma. Vitamin B12 and folic acid as premedication can reduce the frequency of severe toxicities of pemetrexed chemotherapy. However, adverse effects are frequent in clinical settings. In this study, we aimed to identify the clinical factors and single-nucleotide polymorphisms (SNPs) associated with the toxicity and efficacy of pemetrexed chemotherapy.

**Methods:**

This observational study was conducted from October 2012 to December 2019; we evaluated the toxicities and efficacies of pemetrexed chemotherapy using multivariate logistic or Cox regression analysis. In total, 106 patients received pemetrexed chemotherapy. SNPs were analyzed for four patients with malignant pleural mesothelioma and 67 with non-Sq NSCLC.

**Results:**

The median progression-free survival (PFS) and overall survival of 63 patients with non-Sq NSCLC, excluding four in the adjuvant setting, were 6.8 and 33.3 months, respectively. Per propensity-score-adjusted multivariate Cox analyses, favorable factors for PFS were folic acid level ≥ 9.3 ng/mL before premedication, platinum combination, bevacizumab combination, vitamin B12 level < 1136 pg/mL before chemotherapy, A/A + A/G of *BHMT* (742 G > A), and A/A + A/C of *DHFR* (680 C > A). Favorable prognostic factors included good performance status, low smoking index, body mass index ≥ 20.66 kg/m^2^, folic acid level ≥ 5.55 ng/mL before premedication, higher retinol-binding protein before chemotherapy, and A/G of *MTRR* (66 A > G). Among the 71 patients who were analyzed for SNPs, the frequencies of hematologic toxicities and non-hematologic toxicities in Grades 3–4 were 38% and 36.6%, respectively. Per propensity-score-adjusted multivariate logistic analyses, risk factors for Grades 3–4 hematologic toxicities were vitamin B12 level < 486 pg/mL before premedication, leucocyte count < 6120 /µL before chemotherapy, folic acid level < 15.8 ng/mL before chemotherapy, status with a reduced dose of chemotherapy, and C/T + T/T of *MTHFR* (677 C > T). Risk factors for Grades 2–4 non-hematologic toxicities were homocysteine levels ≥ 11.8 nmol/mL before premedication, transthyretin level < 21.5 mg/dL before chemotherapy, C/C + T/T of *MTHFR* (677 C > T), and A/A + G/G of *SLC19A1* [IVS2 (4935) G > A].

**Conclusion:**

The information on metabolites and SNPs of the folate and methionine cycle will help predict the toxicities and efficacies of pemetrexed.

**Trial registration:**

This trial was retrospectively registered with the University hospital Medical Information Network (UMIN000009366) on November 20, 2012.

**Supplementary Information:**

The online version contains supplementary material available at 10.1186/s12885-023-11257-8.

## Background

Pemetrexed is an effective multi-targeted antifolate with acceptable toxicities used to treat non-squamous non-small cell lung cancer (non-Sq NSCLC) and malignant pleural mesothelioma (MPM) [1]. The standard of care for lung cancer includes cytotoxic chemotherapy, targeted therapies for actionable driver mutations, and immuno-oncological therapy. However, no new cytotoxic agents have been developed since 2012. Therefore, the recommended chemotherapeutic regimens involve platinum agents, taxanes, vinorelbine, etoposide, pemetrexed, and gemcitabine [2]. Pemetrexed-based chemotherapies are recommended for MPM [3]. Pemetrexed continues to be a key cytotoxic drug for non-Sq NSCLC and MPM.

As serum homocysteine (Hcy) and methylmalonic acid levels were identified as predictive factors for severe toxicity caused by pemetrexed, patients now receive vitamin B12 (B12) and folic acid (FA) supplementation before pemetrexed-containing chemotherapy [4]. However, even when using B12 and FA as premedication, patients sometimes experience severe toxicities in clinical settings [5–7]. We conducted a retrospective cohort study to evaluate the factors related to pemetrexed toxicities in chemotherapy-naïve patients with non-Sq NSCLC [8]. The predictors of pemetrexed toxicities were poor Eastern Cooperative Oncology Group performance status (ECOG-PS) and low body mass index (BMI) for febrile neutropenia (FN), concomitant chronic infectious disease, bevacizumab use for neutropenia, poor ECOG-PS for thrombocytopenia, and low serum albumin levels for non-hematologic toxicities. Based on the results of a previous study, to reduce unexpected severe toxicities, we comprehensively evaluated Hcy, B12, and FA levels and other clinical features, such as non-hematological or nutritional markers and genetic features.

Previous pharmacogenetic reports dealing with single-nucleotide polymorphisms (SNPs) related to pemetrexed toxicity and efficacy have mainly focused on the transport and metabolism systems [9, 10], folate cycle, and thymidylate cycle [11, 12]. We considered it critical to add SNPs of Hcy-related enzymes to the methionine cycle considering their toxicity. Hence, this prospective study aimed to identify clinical factors and SNPs associated with the toxicities and efficacies of pemetrexed used in chemotherapies.

## Methods

### Patient selection

The inclusion criteria for the patients were as follows: patients 1) with histologically or cytologically proven non-Sq NSCLC and MPM, 2) planning to use pemetrexed-containing chemotherapy, 3) naïve or previously treated with chemotherapy, 4) with ECOG-PS of 0–2, 5) age ≥ 20 years, 6) with adequate organ function measured as leukocyte count ≥ 3,000 /μL, hemoglobin concentration ≥ 10.0 g/dL, platelet count ≥ 100,000 /μL, total bilirubin level ≤ 2.0 mg/dL, transaminase levels ≤ 100 IU/L, serum creatinine level ≤ 1.5 mg/dL, SpO_2_ (percutaneous oxygen saturation) ≥ 90% in ambient air, 7) expected to survive at least 12 weeks from the start of treatment, and 8) who provided written informed consent.

The exclusion criteria were as follows: 1) contraindication to pemetrexed; 2) active concomitant malignancy; 3) uncontrolled comorbidity related to the following systems: respiratory (bronchial asthma, chronic obstructive lung disease, interstitial pneumonia, pulmonary fibrosis, and respiratory failure), cardiac (cardiac failure and arrhythmias), renal (renal failure with dialysis), neurological (cerebral vascular disorder), hepatic disease (Child classification C with liver cirrhosis, fulminant hepatitis, and liver failure); 4) symptomatic brain metastases; 5) active infectious disease; 6) pregnancy or lactation; 7) massive pleural effusion; 8) continuous systemic administration of steroids for comorbid diseases; 9) acute myocardial ischemia within 6 months or unstable angina pectoris; and 10) any other condition judged by the medical oncologist as rendering the patient unsuitable for inclusion.

### Study design

The patients underwent blood tests before premedication administration with vitamin supplements to mitigate toxicity at the start and end of the first cycle. We evaluated adverse events (AEs) and responses during the two cycles. Premedication consisted of B12 and FA; oral administration of FA (500 μg/day) was continued from 7 days before the first dose of pemetrexed until 21 days after the last dose of pemetrexed, whereas 1000 μg B12 was administered intramuscularly, 1 week before the first dose of pemetrexed and every three cycles thereafter. Subsequently, B12 may be injected on the same day as pemetrexed treatment. According to routine clinical practice, which was the same as the prescribing information [13], patients received 500 mg/m^2^ pemetrexed intravenously on day 1 of each 21-day cycle, with or without other antineoplastic agents [14]. If each physician deemed it necessary to prescribe a reduced dose of chemotherapy according to clinical manifestations such as age, ECOG-PS, and modestly impaired organ function, it was acceptable for patients to be administered reduced doses even from the first cycle. The patients continued to receive chemotherapy until progressive disease (PD) [15] or unacceptable toxicity occurred. We also investigated progression-free (PFS) and overall survival (OS).

The primary endpoint of analyses was to determine the relationship between clinical factors or SNPs and toxicities of pemetrexed-containing chemotherapy. The secondary endpoint was identifying the relationship between clinical factors or SNPs and chemotherapy efficacy.

### Evaluation

Before premedication administration, several clinical parameters, such as serum Hcy, B12, FA, prothrombin time (PT), activated partial thromboplastin time (aPTT), and fibrinogen, were investigated. At the start and end of the first cycle, clinical investigations of serum Hcy, serum B12, serum FA, PT, aPTT, fibrinogen, transferrin (Tf), transthyretin (TTR, pre-albumin), retinol-binding protein (RBP), complete blood cell count, and blood chemistry were performed.

Pretreatment evaluation included the following factors: complete medical history, assessment of ECOG-PS, chest X-rays, computed tomography (CT) of the chest to the pelvis, magnetic resonance imaging of the brain, and whole-body bone scintigraphy or 18-fluorodeoxyglucose on Positron Emission Tomography-CT. CT scanning was conducted after two cycles of pemetrexed-containing chemotherapy. According to the Response Evaluation Criteria in Solid Tumors 1.1 [15], tumor lesions were assessed at baseline, every two cycles until 6 months, and then every 2–4 cycles after 6 months until PD. Treatment-related AEs were assessed per the National Cancer Institute Common Terminology Criteria version 4.0 [16]. PFS was defined as the time from the start of pemetrexed-containing chemotherapy to earlier disease progression or death from any cause. OS was defined as the time from the beginning of pemetrexed-containing chemotherapy to death for any reason.

### Pharmacogenetic studies

#### Blood sampling and DNA extraction

First*,* 5 mL of blood was collected from a peripheral vein in EDTA tubes and stored at 4 °C for analysis. DNA was extracted, batched, and genotyped at the Molecular Genetic Analysis Department of the Advanced Technology Center of LSI Medience Corporation (Tokyo, Japan). Briefly, genomic DNA was extracted from the leukocytes using a DNA Blood Mini Kit (Qiagen K.K.-Japan, Tokyo, Japan). All genotyping was polymerase chain reaction (PCR) based. PCR products were prepared for each gene or amplicon and sequenced using the Sanger sequencing method with a BigDye™ Terminator v3.1 Cycle Sequencing Kit and PRISM3130xl Genetic Analyzer (Applied Biosystems; Thermo Fisher Scientific K.K., Tokyo, Japan). SNPs were identified by visual inspection of the gene sequences targeted for this analysis.

#### Selection of SNPs

We examined 17 SNPs in 11 genes (Table 1). Each SNP information is available from the id number of dbSNP on the National Center for Biotechnology Information (NCBI) repository (https://www.ncbi.nlm.nih.gov/snp/). The genetic datasets generated and/or analyzed during the current study are provided in the SNPs data of the Additional file 1. Table 1 includes this SNPs information and a summary of our SNPs dataset in the current study. SNPs in three genes, namely, folylpoly-γ-glutamate synthase (*FPGS*), γ-glutamyl hydrolase (*GGH*), and folate carrier (*SLC19A1*), have been reported as potential predictors of toxicities and efficacy after therapy with pemetrexed [9, 10, 17]. Additionally, SNPs in seven genes, namely, thymidylate synthase (*TYMS*), methionine synthase (*MTR*), methionine synthase reductase (*MTRR*), methylenetetrahydrofolate reductase (*MTHFR*), dihydrofolate reductase (*DHFR*), cystathionine beta-synthase (*CBS*), and betaine-homocysteine methyltransferase (*BHMT*), are associated with the folate and methionine cycle [11, 12, 18–21]. SNPs in the 3-hydroxyisobutyryl-CoA hydrolase (*HIBCH*) gene are related to valine catabolism and useful in diagnosing cobalamin deficiency [22].

**Table 1:** Single-nucleotide polymorphisms (SNPs) information

	**Gene name**	**Protein name**	**SNP location /genotype**	**dbSNP id**	**Genotype (Frequency, %) (*N* = 71)**			**Hetero-zygosity**	**Minor allele frequency**	**Hardy**–**Weinberg equilibrium (*N* = 71)**			**Linkage disequilibrium (*N* = 71)**			
					**Wild type**	**Hetero-zygous**	**Variant**			**Chi-square**	***P*-value (Chi-square)**	***P*-value (Fisher’s exact test)**	**D**	**D’**	**Cor-relation**	**r**^**2**^
1	***FPGS_a***	Folylpoly-γ-glutamate synthase	5'UTR (-63) G > A	rs10760502	GG (95.8)	GA (4.2)	AA (0.0)	0.042	0.02	0.033	1	1	0.014	0.995	0.205	0.042
2	***FPGS_b***	Folylpoly-γ-glutamate synthase	2572 C > T	rs1544105	TT (43.6)	CT (45.1)	CC (11.3)	0.451	0.34	0.004	1	1				
3	***GGH_a***	γ-Glutamyl hydrolase	IVS5 (1042) T > C	rs3780126	CC (35.2)	CT (53.5)	TT (11.3)	0.475	0.38	1.304	0.317	0.320	NA	NA	NA	NA
4	***GGH_b***	γ-Glutamyl hydrolase	IVS1 (1307) C > A	rs7010484	AA (100)	CA (0.0)	CC (0.0)	0	0	NA	NA	NA				
5	***SLC19A1_a***	Folate carrier (SLC19A1)	IVS2 (4935) G > A	rs914232	AA (21.1)	GA (61.9)	GG (17.0)	0.503	0.48	4.146	0.059	0.059	0.238	0.999	0.958	0.918
6	***SLC19A1_b***	Folate carrier (SLC19A1)	Exon 6 (2522) C > T (3'UTR)	rs1051298	TT (23.9)	CT (60.6)	CC (15.5)	0.499	0.46	3.436	0.055	0.095				
7	***MTRR***	Methionine synthase reductase	66 A > G	rs1801394	AA (43.7)	AG (50.7)	GG (5.6)	0.431	0.31	2.444	0.175	0.168				
8	***MTR***	Methionine synthase	2756 A > G	rs1805087	AA (64.8)	AG (32.4)	GG (2.8)	0.310	0.19	0.191	0.699	1				
9	***MTHFR_a***	Methylenetetrahydrofolate reductase	677 C > T	rs1801133	CC (38.0)	CT (50.7)	TT (11.3)	0.467	0.37	0.605	0.461	0.609	-0.064	0.998	-0.351	0.123
10	***MTHFR_b***	Methylenetetrahydrofolate reductase	1298 A > C	rs1801131	AA (67.6)	AC (29.6)	CC (2.8)	0.292	0.18	0.027	1	1				
11	***DHFR***	Dihydrofolate reductase	680 C > A	rs442767	AA (33.8)	CA (52.1)	CC (14.1)	0.484	0.4	0.506	0.462	0.622				
12	***CBS_a***	Cystathionine beta synthase	833 T > C	rs5742905	TT (100)	TC (0.0)	CC (0.0)	0	0	NA	NA	NA	NA	NA	NA	NA
13	***CBS_b***	Cystathionine beta synthase	844 ins68	rs863223428	ins; not detected			0	0	NA	NA	NA				
14	***CBS_c***	Cystathionine beta synthase	919 G > A	rs121964962	GG (100)	GA (0.0)	AA (0.0)	0	0	NA	NA	NA				
15	***HIBCH***	3-Hydroxyisobutyryl-CoA hydrolase	c.2 T > C p.Met1	rs291466	TT (64.8)	TC (35.2)	CC (0.0)	0.292	0.18	3.242	0.028	0.107				
16	***BHMT***	Betaine-homocysteine methyltransferase	742 G > A	rs3733890	GG (62.0)	GA (31.0)	AA (7.0)	0.352	0.23	0.899	0.491	0.320				
17	***TYMS***	Thymidylate synthase	VNTR in 5′UTR	rs749847423	2R2R(2.8), 2R3R(32.4), 3R3R(63.4), 3R5R(1.4)			-	-	NA	NA	NA				

*Abbreviations*: *dbSNP id* Id number of dbSNP on the National Center for Biotechnology Information (NCBI) repository [https://www.ncbi.nlm.nih.gov/snp/], *VNTR* The variable number of tandem repeats, *UTR* Untranslated regions, *IVS* Instrumental variables, *ins* Insertion, *NA* Not applicable

### Statistical analyses

The target sample size was calculated as follows. We aimed to detect more than two significant risk factors for Grade (G) 4 neutropenia in this study. When 4% of patients treated with pemetrexed-containing chemotherapy would experience G4 neutropenia, the accrual of at least 50 patients was needed, assuming that a significant factor of 20 to 25 would be identified for the outcome in the multivariate logistic regression analysis, in general. When we consider a 20% dropout rate, at least 60 patients without missing values would be required.

For continuous variables in efficacy analyses, receiver operating characteristic (ROC) curves were analyzed using the SigmaPlot software version 14.5 (Systat Software, Inc., San Jose, CA, USA) [23]. In the toxicity analyses, the median was used to dichotomize continuous variables. Logistic regression analysis was performed to assess risk factors for AEs in the 1st cycle. PFS and OS were estimated using the Kaplan–Meier method. Cox regression analysis was performed to investigate favorable factors for PFS and OS.

We performed crude and propensity score (PS)-adjusted analyses to reduce potential confounding and bias in the observational studies [24]. We constructed a PS for the median Hcy level at the start of the first cycle of chemotherapy using a logistic regression model for each patient for both toxicity and efficacy evaluation. The PS for adjustment of toxicities was composed using body surface area, age, histology, relapse after surgical resection, ECOG-PS, comorbidities, chemotherapy line, reduced dosage requirement status, and duration of premedication as independent variables, and the C-statistic of the ROC curve analysis was 0.808. The PS for the adjustment of efficacy constituted the following variables: independent variables such as bevacizumab combination, chemotherapeutic regimen, relapses after radical radiotherapy or surgical resection, ECOG-PS, driver mutation, BMI, chemotherapy line, reduced dosage requirement status, duration of premedication, and comorbidities (respiratory, gastrointestinal, neuropsychiatric, urologic-renal, and dermato-orthopedic), with a C-statistic of 0.823.

We performed model selection for multivariate analysis according to a previously described method [23, 24]. Briefly, we identified variables with significance at *P* < 0.1 in the univariate analysis. Spearman’s rank test and clinically clarified dependent variables were used to exclude dependent variables from the selected variables. A correlation coefficient (rho) of > 0.3 as the absolute value with the Spearman’s rank test indicated a significant association. When many variables were detected in univariate analyses, we used rho > 0.2 at the absolute value as a significant association to converge the parameters correctly. Models were constructed using only independent variables as candidates. Akaike’s Information Criterion (AIC) was used to select the best model from among the candidate models. After choosing the best model, we proceeded with stepwise variable deletion to minimize the AIC. The final model was composed only of variables that achieved the minimum AIC during the elimination process. In the final multivariate analysis using a simultaneous method, statistical significance was determined at *P* < 0.05 with a two-sided test. Moreover, a sensitivity analysis was conducted using a model with one less variable than that of the final model.

All analyses were performed using the SPSS Statistics software version 27 (IBM, New York, USA). We used the “Genetics: Population Genetics” package version 1.3.8.1.3 developed by Gregory Warnes in R version 4.2.1 to calculate the Hardy–Weinberg equilibrium and linkage disequilibrium (LD), expressed as D′ and r^2^ [25].

## Results

### Patients

**Fig. 1:**
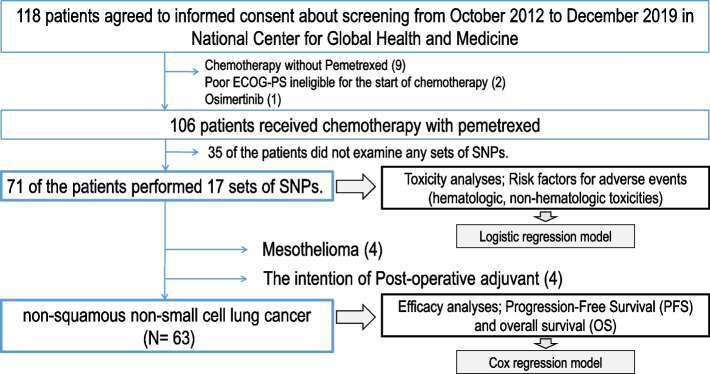
Flow chart of the accrual of patients and the performed analyses. Patients were accrued, and analyses were performed according to the flow chart

From October 2012 to December 2019, 118 patients provided informed consent for the observational cohort study (Fig. 1). Of these, nine received chemotherapy without pemetrexed, and one patient received osimertinib. ECOG-PS of two patients did not meet the criteria before chemotherapy. Thirty-five could not agree to the SNP examination even though they agreed to participate in the observational cohort study for treatment. Toxicity was analyzed in 71 patients. Seventeen SNPs were combined with clinical and metabolite information for this examination (Table 1). Excluding mesothelioma and postoperative adjuvant chemotherapy, 63 patients had non-Sq NSCLC with stage IIIB to IV (UICC ver.8) or recurrence after surgical treatment for efficacy analyses.

The key patient characteristics are summarized in Table 2. The median age of the patients was 72 years, and 22 female patients were included. Ninety-six percent of the patients had an ECOG-PS of 0 to 1, the median smoking index (SI) was 440, and 24 reported never smoking. Histologically, 67 patients had non-Sq NSCLC and four had MPM. Comorbidities were noted in 61 patients. The median BMI was 21.7 kg/m^2^. Fifty-five were chemotherapy-naïve patients. Sixty patients received platinum combined with pemetrexed. Eighteen patients with non-sq NSCLC were treated in combination with bevacizumab and seven with pembrolizumab. Twelve patients were treated with a reduced dose of pemetrexed in the first cycle. The median duration of premedication was 11 days. We also evaluated nutrition testing. The median Tf, TTR, and RBP levels were normal.

**Table 2:** Key Background Characteristics of patients who performed SNPs testing (*n*=71)

**Characteristics**		**Number (%) or Median (Range)**	**Clinical Investigation**		**Median (Range)**
**Age (years)**		72 (36-85)	**Premedication**		
**Gender**	Male	49 (69)	Duration (Days)		11 (6 – 42)
	Female	22 (31)	serum Homocysteine	Before premedication	11.8 (6.5 – 35.3)
**ECOG performance status**			(nmol/mL)	Before chemotherapy	10 (5.8 – 22.4)
	0	30 (42)	serum Vitamin B12	Before premedication	486 (195 – 2960)
	1	38 (54)	(pg/mL)	Before chemotherapy	664 (259 – 5080)
	2	3 (4)	serum Folic acid	Before premedication	6.9 (1.6 – 22.3)
**Smoking Index**		440 (0 – 1800)	(ng/mL)	Before chemotherapy	15.8 (6.9 – 81.2)
**Clinical stage (Ver. 8)**			**Hematology before chemotherapy**		
	I to IIIB	25 (35)	White blood cell (WBC) count (/µL)		6,120 (3,540 – 21,430)
	IIIC to IVB	46 (65)	Neutrophil count (/µL)		4,126 (1,750 – 19,223)
**Histology**	NSCLC; Adenocarcinoma / Other	64 (90) / 3 (4)	Monocyte count (/µL)		401 (61 – 1,431)
	Mesothelioma	4 (6)	Lymphocyte count (/µL)		1153 (86 – 2,727)
**TTF1**	Negative (-)		Red blood cell (RBC) count (x 10^4^/µL)		400 (285 – 545)
	Faint (±)	5 (7)	Hemoglobin concentration (g/dL)		12.1 (7.9 – 17.4)
	Positive (+)	43 (60)	Thrombocyte count (x 10^4^/µL)		23.8 (9 – 62.6)
	unexamined	14 (20)	**Non-Hematology before chemotherapy**		
**Driver Mutation**			AST, aspartate aminotransferase (U/L)		21 (9 – 76)
	Negative	45 (63)	ALT, alanine aminotransferase (U/L)		16 (5 – 103)
	Positive	26 (37)	Albumin (g/dL)		3.7 (2.2 – 4.9)
			ChE, Cholinesterase (U/L)		249 (76 – 559)
**Comorbidities**			BUN, Blood urea nitrogen (mg/dL)		14.1 (5.1 – 27.1)
	Absent	10 (14)	Creatinine (mg/dL)		0.73 (0.36 – 1.39)
	Present	61 (86)	eGFR, estimated glomerular filtration rate (mL/min)		69.4 (38.5 – 128.5)
**Body Mass Index (BMI) (kg/m**^**2**^**)**		21.7 (15.2 – 34.7)	CRP, C-reactive protein (mg/dL)		0.54 (0.002 – 24.5)
**Chemotherapy**			PT-INR, international normalized ratio of prothrombin time		1.00 (0.79 – 1.96)
	Line; Adjuvant / 1 / 2 & more	4 (6) / 51 (72) / 16 (22)	APTT, activated part Thromboplastin time (second)		29 (21.2 – 46)
	Mono / Platinum-doublet	11 (15) / 60 (85)	Fibrinogen (mg/dL)		401 (241 – 1015)
	without/with Bevacizumab	53 (75) / 18 (25)	** Nutrition testing**		
	without/with Pembrolizumab	64 (90) / 7 (10)	Tf, Transferrin (mg/dL)		229 (110 – 346)
	Dose reduction from the initial cycle:		TTR, Transthyretin (mg/dL)		21.5 (4 – 42)
	Absent / Present	59 (83) / 12 (17)	RBP, Retinol binding protein (mg/dL)		2.95 (0.8 – 6.3)

### Genetic polymorphisms

Table 1 lists the SNPs examined in this study. Twelve of the seventeen SNPs were heterozygous. The median minor allele frequency was 0.33, with a range of 0.02–0.48. Their *P*-values via Fisher’s exact tests were greater than 0.05, indicating that they were in the Hardy–Weinberg equilibrium. A weak LD was suggested between *FPGS_a* and *FPGS_b* (D’ = 0.995 and r^2^ = 0.042). Moderate LD was suggested between *MTHFR_a* and *MTHFR_b* (D’ = 0.998 and r^2^ = 0.123, respectively). A strong LD was suggested between *SLC19A1_a* and *SLC19A1_b* (D’ = 0.999 and r^2^ = 0.918).

### Toxicity analyses

Seventy-one patients underwent toxicity analysis (Fig. 1). When patients experience severe toxicities in the initial cycle of cytotoxic chemotherapy, they are given a reduced dose to tolerate toxicities in subsequent cycles [13]. Therefore, we used AEs during the first cycle as toxicities in this study (Table 3 and Supplementary Table S1 in Additional file 2). Among all causalities, 58% were scored > G3. Hematological AEs > G3 constituted 38%. G3–G4 hematologic toxicities were neutropenia (31.0%), leukopenia (18.3%), thrombocytopenia (14.1%), and anemia (9.9%) (Tables 3 and S1). Non-hematological AEs > G3 constituted 37% of the AEs. Common G2–G4 non-hematologic toxicities were anorexia (39.4%), serum alanine aminotransferase (ALT) level elevation (19.7%), FN (18.3%), and nausea (14.1%) (Table S1).

**Table 3:** Adverse events during the 1st cycle of pemetrexed-containing chemotherapy

**Adverse event**	Grade (CTCAE), n (%) *N* = 71		
	3	4	≥ 3
**All-causality AE**	32 (45.0)	9 (12.7)	41 (57.7)
** Hematological AE**	20 (28.2)	7 (9.9)	27 (38.0)
Leukopenia	12 (16.9)	1 (1.4)	13 (18.3)
Neutropenia	15 (21.1)	7 (9.9)	22 (31.0)
Anemia	7 (9.9)	0 (0)	7 (9.9)
Thrombocytopenia	6 (8.5)	4 (5.6)	10 (14.1)
**Non-hematological AE**	23 (32.4)	3 (4.2)	26 (36.6)
Febrile neutropenia	12 (16.9)	1 (1.4)	13 (18.3)
AST (aspartate aminotransferase)	2 (2.8)	1 (1.4)	3 (4.2)
ALT (alanine aminotransferase)	8 (11.3)	0 (0)	8 (11.3)
Anorexia	1 (1.4)	0 (0)	1 (1.4)
Skin disorders	1 (1.4)	0 (0)	1 (1.4)
Infection	5 (7.0)	0 (0)	5 (7.0)
Others (Intestinal perforation)	0 (0)	1 (1.4)	1 (1.4)

*CTCAE ver. 4.0* Common terminology criteria for adverse events, *AE* Adverse event

**Fig. 2:**
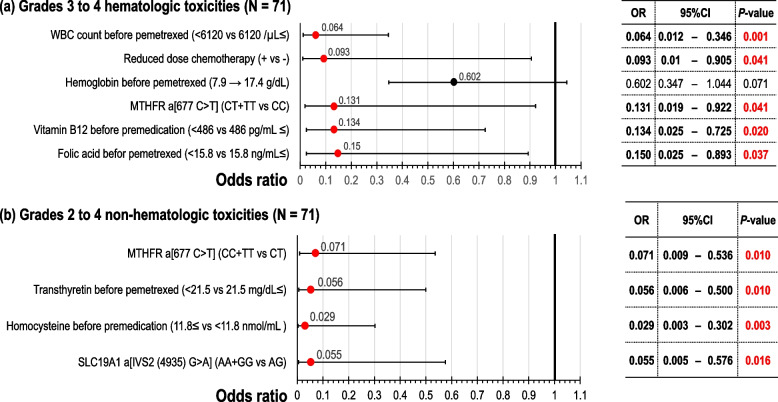
Propensity score-adjusted risk factors for adverse events in the first cycle using multivariate logistic regression analysis. Variables with a *P*-value < 0.10 on univariate analysis were entered into multivariate logistic analysis using a simultaneous method. **a** Grades 3–4 hematologic toxicities (*N* = 71). **b** Grades 2–4 non-hematologic toxicities (*N* = 71) *WBC* white blood cell

Multivariate logistic regression analysis revealed that crude low-risk factors for G3–G4 hematological AEs in the first cycle were high white blood cell (WBC) count before pemetrexed initiation, adapted status for using standard dosage, and C/C genotype group of *MTHFR_a* [677 C > T] (Supplementary Table S2(a)). PS-adjusted low-risk factors for G3–G4 hematological AEs were associated with WBC count ≥ 6120 /μL before pemetrexed exposure [odds ratio (OR): 0.06; 95% confidence interval (CI): 0.01–0.35; *P* = 0.001], adapted status for using standard dosage [OR: 0.09; 95% CI: 0.01–0.91; *P* = 0.04], C/C genotype group of *MTHFR_a* [OR: 0.13; 95% CI: 0.02–0.92; *P* = 0.04], serum B12 level ≥ 486 pg/mL before premedication [OR: 0.13; 95% CI: 0.03–0.73; *P* = 0.02], and serum FA level ≥ 15.8 ng/mL before pemetrexed exposure [OR: 0.15; 95% CI: 0.03–0.89; *P* = 0.04] (Supplementary Table S2(b) and Fig. 2a).

For non-hematological AEs using multivariate logistic regression analysis, the C/T genotype group of *MTHFR_a*, high serum RBP level before pemetrexed treatment, high serum B12 level before premedication, and high red blood cell (RBC) count before pemetrexed treatment (Supplementary Table S3(a)) were the crude low-risk factors for G2–G4 non-hematological AEs in the first cycle. After adjusting for PS, four variables constituted the significant low-risk factors for G2–G4 non-hematological AEs: C/T genotype group of *MTHFR_a* [OR: 0.07; 95% CI: 0.009–0.54; *P* = 0.01], TTR level ≥ 21.5 mg/dL before pemetrexed treatment [OR: 0.06, 95% CI: 0.006–0.50; *P* = 0.01], serum Hcy level < 11.8 nmol/mL before premedication [OR: 0.03; 95% CI: 0.003–0.30; *P* = 0.003], and A/G genotype group of *SLC19A1_a* [IVS2(4935) G > A, OR: 0.06; 95% CI: 0.005–0.58; *P* = 0.02] (Supplementary Table S3(b) and Fig. 2b).

In the case of 14% or more G3–G4 hematological AEs or G2–G4 non-hematological AEs (Supplementary Table S1), we examined the risk factors for each AE. All these risk factors were analyzed using multivariate logistic regression analysis adjusted for PS (Supplement Fig. S1 in Additional file 3). Adapted status for using standard dosage [OR: 0.03; 95% CI: 0.002–0.37; *P* = 0.007] (Supplement Fig. S1(a)) served as the sole significant low-risk factor for G3–G4 leucopenia. A WBC count ≥ 6120/μL before pemetrexed exposure [OR, 0.05; 95% CI: 0.008–0.35; *P* = 0.002] was a low-risk factor for G3–G4 neutropenia (Supplement Fig. S1(b)). Higher hemoglobin levels before pemetrexed treatment [OR: 0.03; 95% CI: 0.002–0.33; *P* = 0.005] and lower Hcy levels before pemetrexed treatment [OR: 0.58; 95% CI: 0.37–0.90; *P* = 0.02] were low-risk factors for G2–G4 anemia (Supplement Fig. S1(c)). Low-risk factors for G3–G4 thrombocytopenia were as follows: a higher FA level before pemetrexed exposure [OR: 0.67; 95% CI: 0.47–0.97; *P* = 0.03], WBC count ≥ 6120 /μL before pemetrexed exposure [OR: 0.009; 95% CI: 0.0001–0.71; *P* = 0.03], and C/C or T/T genotype groups of *MTHFR_a* [OR: 0.05; 95% CI: 0.003–0.73; *P* = 0.03] (Supplement Fig. S1(d)). Low-risk factors for G2–G4 ALT elevation included higher FA levels before premedication [OR: 0.45, 95% CI: 0.25–0.83; *P* = 0.01], presence of comorbidities [OR: 0.01, 95% CI: 0.0001–0.61; *P* = 0.03], and a serum ALT level < 16 U/L before pemetrexed exposure [OR: 0.03, 95% CI: 0.002–0.51; *P* = 0.02] (Supplement Fig. S1(e)). Low-risk factors for G2–G4 anorexia included a platelet count < 23.8 × 10^4^ /μL before pemetrexed exposure [OR: 0.22; 95% CI: 0.05–0.98; *P* = 0.046] and serum B12 level ≥ 486 pg/mL before premedication [OR: 0.18, 95% CI: 0.04–0.74; *P* = 0.02] (Supplement Fig. S1(f)). Low-risk factors for G2–G4 nausea were the C/T or C/C genotype groups of *FPGS_b* [2572 C > T, OR: 0.11; 95% CI: 0.01–0.76; *P* = 0.03] and a B12 level ≥ 486 pg/mL before premedication [OR: 0.08, 95% CI: 0.01–0.66; *P* = 0.02] (Supplement Fig. S1(g)). Low-risk factors for G3–G4 FN included adapted status for using standard dosage [OR: 0.006, 95% CI: 0.0003–0.16; *P* = 0.002], RBC count ≥ 400 × 10^4^ /μL before pemetrexed exposure [OR: 0.02, 95% CI: 0.001–0.44; *P* = 0.01], neutrophil count ≥ 4127 /μL before pemetrexed exposure [OR: 0.06; 95% CI: 0.005–0.80; *P* = 0.03], and the A/C genotype group of *DHFR* [680 C > A, OR: 0.02; 95% CI: 0.0007–0.39; *P* = 0.01] (Supplement Fig. S1(h)). We performed sensitivity analyses for all the above final models, and each sensitivity analysis demonstrated the same tendency as the corresponding final model.

### Efficacy analyses

**Fig. 3:**
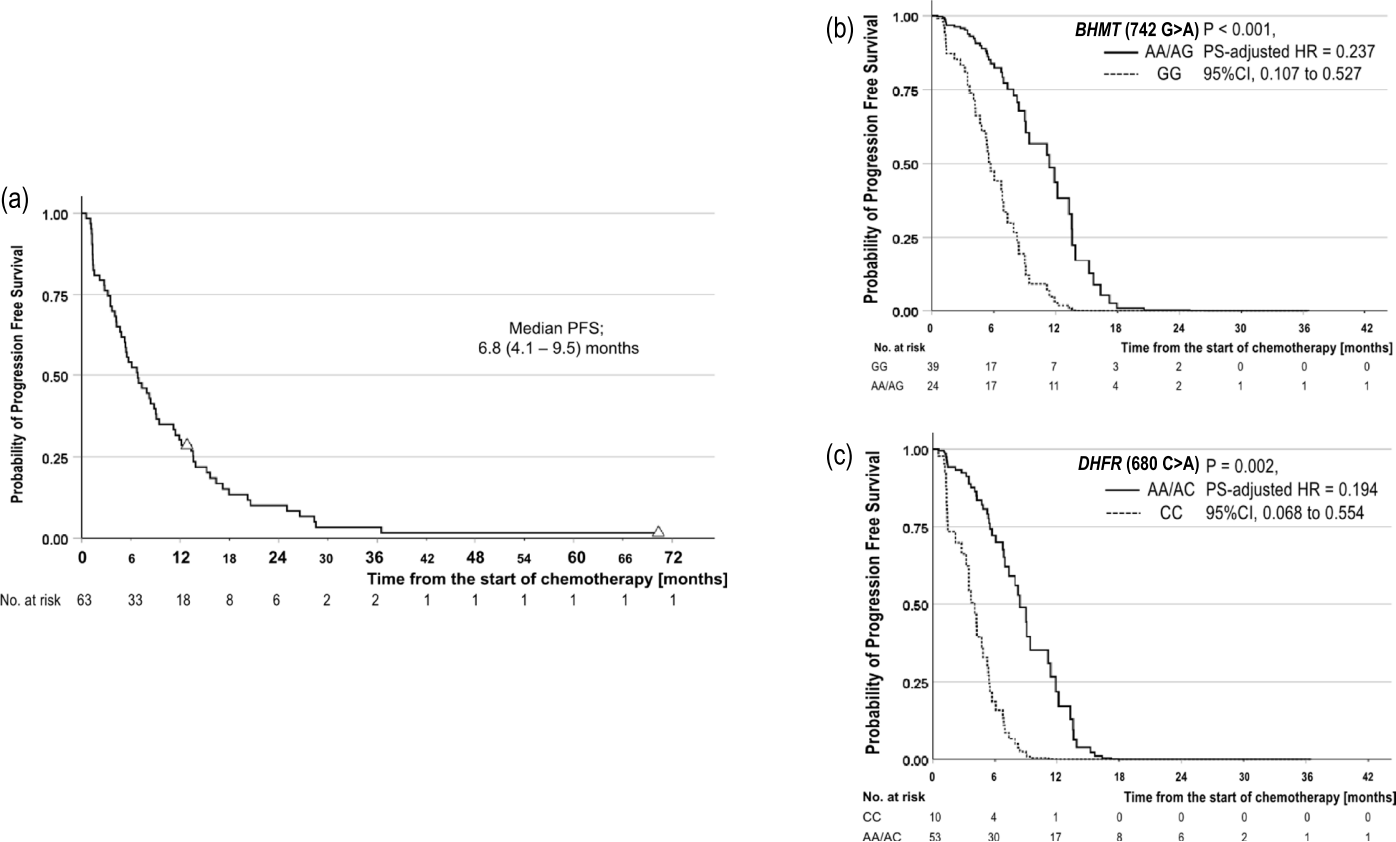
Progression-free survival (PFS) curve in patients with NSCLC who underwent SNP analyses. **a** PFS curve of total patients with NSCLC using the Kaplan–Meier method (*N* = 63). **b** PFS curves of patients with the GG genotype group (Short dash) and the AA + AG genotype group (Solid) of BHMT (742 G > A) using propensity score (PS)-adjusted multivariate Cox regression analyses. **c** PFS curves of patients with the CC genotype group (Short dash) and the AA + AC genotype group (Solid) of DHFR (680 C > A) using PS-adjusted multivariate Cox regression analyses

**Fig. 4:**
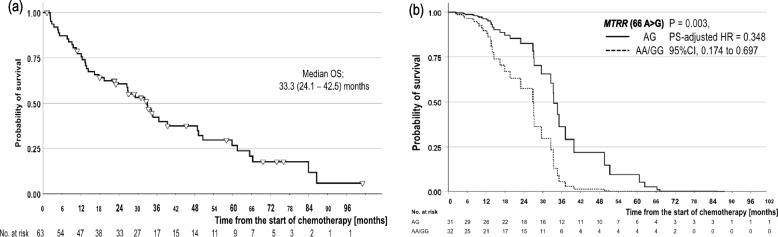
Overall survival (OS) curve of patients with NSCLC who underwent SNP analyses. **a** OS curve of total patients with NSCLC using the Kaplan–Meier method (*N* = 63). **b** OS curves of patients with the AA + GG genotype group (Short dash) and the AG genotype group (Solid) of MTRR (66 A > G) using propensity score (PS)-adjusted multivariate Cox regression analyses

The efficacy analyses for non-Sq NSCLC included 63 patients (Fig. 1). The objective response rate was 28.2%. The final survival assessment was conducted on September 30, 2021. The median PFS was 6.8 months, and its 95% CI ranged from 4.1 to 9.5 (Fig. 3a). The median OS was 33.3 months, ranging from 24.1 to 42.5 (Fig. 4a).

**Fig. 5:**
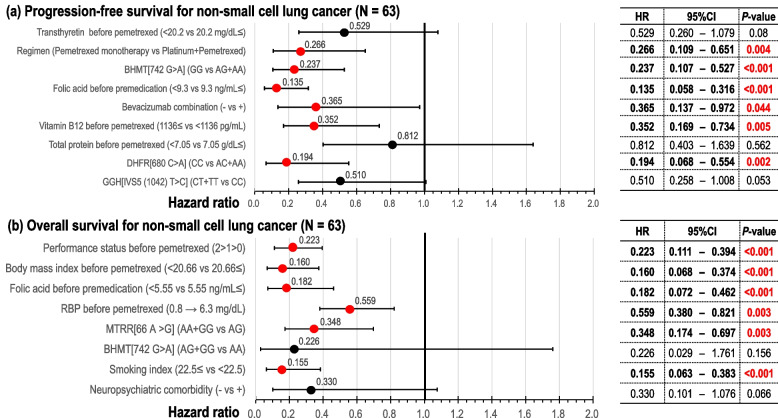
Propensity score-adjusted efficacies using multivariate Cox regression analyses. Variables with a *P*-value < 0.10 on univariate analysis were entered into multivariate Cox analysis by a simultaneous method. **a** Progression-free survival (*N* = 63). **b** Overall survival (*N* = 63) *RBP* retinol-binding protein

We conducted crude and PS-adjusted multivariate Cox regression analyses to identify the factors favorable for PFS. From crude multivariate analyses, pemetrexed combined with platinum, regimen combined with bevacizumab, B12 level < 1136 pg/mL before pemetrexed treatment, FA level ≥ 9.3 ng/mL before premedication, A/G or A/A genotype group of *BHMT* (742 G > A), and neutrophil to lymphocyte ratio < 3.51 before pemetrexed treatment were identified to be the favorable factors for PFS (Supplementary Table S4(a)). As the final result of PS-adjusted multivariate analyses, the favorable factors for PFS were pemetrexed combined with the platinum [hazard ratio (HR): 0.27; 95% CI: 0.11–0.65; *P* = 0.004], the A/G or A/A genotype group of *BHMT* (742 G > A) (HR: 0.24; 95% CI: 0.11–0.53; *P* < 0.001, Fig. 3b), FA levels ≥ 9.3 ng/mL before premedication [HR: 0.14; 95% CI: 0.06–0.32; *P* < 0.001], a regimen combined with bevacizumab [HR: 0.37; 95% CI: 0.14–0.97; *P* = 0.04], B12 level < 1136 pg/mL before pemetrexed treatment [HR: 0.35; 95% CI: 0.17–0.73; *P* = 0.005], and the A/C or A/A genotype group of *DHFR* (680 C > A) (HR: 0.19; 95% CI: 0.07–0.55; *P* = 0.002, Fig. 3c) (Supplementary Table S4(b) and Fig. 5a). The sensitivity analysis demonstrated the same tendency as that of the final model.

In the multivariate Cox regression analyses, crude favorable prognostic factors included a good ECOG-PS before pemetrexed exposure, BMI ≥ 20.66, FA level ≥ 5.55 ng/mL before premedication, higher serum RBP level before pemetrexed treatment, SI < 22.5, and the A/G genotype group of *MTRR* (66 A > G) (Supplementary Table S5(a)). After adjusting for PS, the significantly favorable prognostic factors were improved ECOG-PS [HR: 0.22; 95% CI: 0.11–0.39; *P* < 0.001], a BMI ≥ 20.66 kg/m^2^ [HR: 0.16; 95% CI: 0.07–0.37; *P* < 0.001], an FA level ≥ 5.55 ng/mL before premedication [HR: 0.18; 95% CI: 0.07–0.46; *P* < 0.001], a higher RBP level before pemetrexed treatment [HR: 0.56; 95% CI: 0.38–0.82; *P* = 0.003], an SI < 22.5 [HR: 0.16; 95% CI: 0.06–0.38; *P* < 0.001], and the A/G genotype group of *MTRR* (66 A > G) (HR: 0.35; 95% CI: 0.17–0.70; *P* = 0.003, Fig. 4b) (Supplementary Table S5(b) and Fig. 5b). The sensitivity analysis demonstrated the same trend as that of the final model.

## Discussion

This study explored and evaluated the factors that contribute to the AEs of pemetrexed and those that are related to its efficacy based on clinical background, laboratory tests, and pharmacogenetic factors. Regarding the toxicity of pemetrexed, low-risk factors for hematological AEs were high WBC count before pemetrexed exposure, the standard dosage of chemotherapeutic agents, *MTHFR_a* polymorphisms, high B12 level before premedication, and a high FA level before pemetrexed exposure (Fig. 2a). Low-risk factors for non-hematological AEs were *MTHFR_a* polymorphisms, high TTR levels before pemetrexed exposure, low Hcy levels before premedication, and *SLC19A1_a* polymorphisms (Fig. 2b). For non-Sq NSCLC, we found that the favorable factors for PFS were platinum plus pemetrexed, *BHMT* polymorphisms (Fig. 3b), a high FA level before premedication, bevacizumab combination, *DHFR* polymorphisms (Fig. 3c), and a low B12 level before pemetrexed treatment (Fig. 5a) and that favorable prognostic factors were good ECOG-PS, a high BMI, a high FA level before premedication, high RBP level before pemetrexed treatment, *MTRR* polymorphisms (Fig. 4b), and low SI (Fig. 5b).

**Fig. 6:**
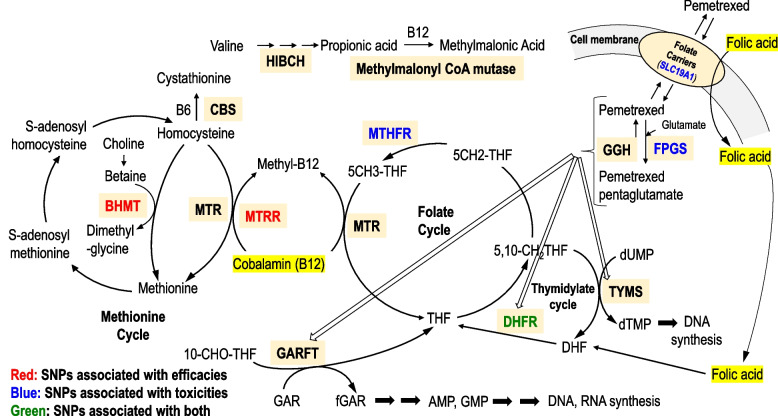
Mechanism of action and associated SNPs of pemetrexed. The text in red indicates SNPs that are related to efficacy. The text in blue indicates SNPs that are related to toxicity. The text in green indicates SNPs that are related to both efficacy and toxicity. The yellow text represents premedication supplements. AMP, adenosine monophosphate; B6, vitamin B6; B12, vitamin B12; BHMT, betaine-homocysteine methyltransferase; CBS, cystathionine beta-synthase; DHF, dihydrofolate; DHFR, dihydrofolate reductase; dUMP, deoxyuridine monophosphate; dTMP, deoxythymidine monophosphate; fGAR, form N^2^-formyl-N^1^-(5-phospho-D-ribosyl)glycinamide; FPGS, folylpoly-γ-glutamate synthase; GAR, N^1^-(5-phospho-D-ribosyl)glycinamide; GARFT, GAR transformylase; GGH, γ-glutamyl hydrolase; GMP, guanosine monophosphate; HIBCH, 3-hydroxyisobutyryl-CoA hydrolase; MTHFR, methylenetetrahydrofolate reductase; MTR, methionine synthase; MTRR, methionine synthase reductase; SLC19A1, folate carrier; THF, tetrahydrofolate; TYMS, thymidylate synthase

The molecular mechanism of pemetrexed and associated SNPs are shown in Fig. 6. We identified pharmacogenetic implications for six polymorphisms. Hcy-related enzyme SNPs in the methionine cycle were associated with efficacy. The MTRR polymorphism is a risk factor for lung cancer incidence [18], and we found that the MTRR polymorphism was associated with the prognosis of lung cancer (Fig. 4b). There are few reports on the relationship between *BHMT* polymorphisms and human disease. *BHMT* polymorphism may play a protective role in the risk of coronary heart disease [26] and is associated with the incidence of uterine cervical cancer [27]. We newly found that the A/G or A/A genotype of *BHMT* (742 G > A) was a favorable factor for PFS in pemetrexed-based chemotherapy (Fig. 3b). *BHMT* polymorphism may predict the efficacy of pemetrexed-containing regimens. Three SNPs (*SLC19A1*, *FPGS*, and *MTHFR*) were associated with toxicity. They have been associated with pemetrexed transport [9], metabolism [17], and the folate cycle [19], respectively. Furthermore, the SNP in *DHFR* (680 C > A) was associated with toxicity and efficacy (Fig. 3c). DHFR has been previously reported to be related to the thymidylate cycle [12] and is the target enzyme of pemetrexed [13]. Notably, by examining these SNPs, it may be possible to predict toxicities and efficacies even before the use of pemetrexed and to identify the beneficial group of pemetrexed as a personalized treatment approach.

As SNPs are associated with the physical constitution of individuals, patients with specific polymorphisms will experience toxicity every time they use pemetrexed. In previous studies, serum Hcy levels have been reported to be primarily associated with hematological AEs in patients receiving pemetrexed with or without premedication [4, 28]. However, in our study, serum Hcy levels were associated with non-hematological AEs and anemia. Additionally, B12 levels before premedication and FA levels before pemetrexed exposure were related to hematological AEs. Appropriate premedication with B12 and FA is considered helpful in preventing severe hematological AEs, consistent with the findings of previous reports [4, 29]. The serum levels of B12 and FA were associated with hematological AEs and efficacy in the multivariate analyses. Therefore, investigating the serum B12 levels before premedication and FA levels before pemetrexed administration may help predict hematological AEs.

This study had several limitations. First, this was a single-center study with a relatively small sample size, despite being a prospective observational study. To shorten the entry period, any pemetrexed-containing chemotherapy and any line of chemotherapy were acceptable for accrual. As the primary endpoint was to evaluate toxicities, both non-Sq NSCLC and MPM were included. However, a long enrollment period was necessary owing to the inclusion of genetic studies. Second, as this study is an ethnically homogeneous cohort of Japanese subjects, we could not examine ethnic differences in terms of toxicities and efficacies. Despite this limitation, the findings are novel and may be used to highlight predictors of toxicities and efficacies before the administration of pemetrexed. The association of toxicities and efficacies with ethnic origin warrants further investigation in the future. Third, although we performed statistical adjustments, the bias of these background factors could not necessarily be eliminated. Fourth, multivariate analyses can identify important predictive factors for efficacy or toxicities. However, the results of this exploratory study must be verified by conducting a confirmatory study.

Nonetheless, this study had several strengths. To date, there have been some SNP studies using univariate analyses. This study identified predictive SNPs for efficacy or toxicity via multivariate analyses. Folate and methionine cycles are generally related to the action of pemetrexed (Fig. 6). We found that the SNPs of the enzymes in these two cycles were associated with toxicities or efficacies, even when using multivariate analyses. Collectively, using the information on the SNPs as biomarkers can help predict efficacy and avoid unexpected heavy toxicities. This can lead to personalized patient care for pemetrexed-containing regimens.

## Conclusions

Among patients under pemetrexed-containing chemotherapy regimens, we found that information on metabolites and enzyme SNPs of the folate, methionine, and thymidylate cycles help predict the toxicity and efficacy of pemetrexed. Particularly, from the perspective of pharmacogenetics, six SNPs were associated with efficacy and toxicity profiles. Risks for G3–G4 hematological toxicities were leucocyte < 6120 /μL before chemotherapy, status with a reduced dose of chemotherapy, C/T + T/T genotype of *MTHFR* (677 C > T), B12 levels < 486 pg/mL before premedication, and FA levels < 15.8 ng/mL before chemotherapy. Risks for G2–G4 non-hematological toxicities were C/C + T/T of *MTHFR* (677 C > T), TTR level < 21.5 mg/dL before chemotherapy, Hcy levels ≥ 11.8 nmol/mL before premedication, and A/A + G/G of *SLC19A1 IVS2 (*4935) G > A. Risks for G2–G4 nausea were T/T of *FPGS* (2572 C > T). Favorable factors for PFS were the platinum combination, A/A + A/G genotype of *BHMT* (742 G > A), FA levels ≥ 9.3 ng/mL before premedication, bevacizumab combination, B12 level < 1136 pg/mL before chemotherapy, and A/A + A/C genotype of *DHFR* (680 C > A). Favorable prognostic factors included good performance status, BMI ≥ 20.66 kg/m^2^, FA levels ≥ 5.55 ng/mL before premedication, higher RBP before chemotherapy, A/G of *MTRR* (66 A > G), and low SI.

Using this information to reduce unexpected AEs will benefit patients receiving pemetrexed-containing chemotherapies. We expect that such information will contribute to the safe and effective use of pemetrexed.

### Supplementary Information

**Additional file 1.**

**Additional file 2: Supplementary Table S1.** All types of adverse events during the 1st cycle of pemetrexed-containing chemotherapy. **Supplementary Table S2.** Risk factors for grades 3 to 4 hematological adverse events at the 1st cycle by logistic regression analysis (*N* = 71). **Supplementary Table S3.** Risk factors for grades 2 to 4 non-hematological adverse events at the 1st cycle by logistic regression analysis (*N* = 71). **Supplementary Table S4.** Progression-free survival for non-squamous non-small cell lung cancer by Cox regression analyses (*N* = 63). **Supplementary Table S5.** Overall survival for non-squamous non-small cell lung cancer by Cox regression analyses (*N* = 63).

**Additional file 3: Supplement Fig S1.** Propensity score-adjusted risk factors for toxicities at the 1st cycle by multivariate logistic regression analysis (*N* = 71).

## Data Availability

The datasets used and/or analyzed during the current study are available from the corresponding author upon reasonable request.

## Abbreviations

95% CI: 95% Confidential interval
AEs: Adverse events
AIC: Akaike’s Information Criterion
ALT: Alanine aminotransferase
aPTT: Activated partial thromboplastin time
BHMT: Betaine-homocysteine methyltransferase
BMI: Body mass index
B12: Vitamin B12
CBS: Cystathionine beta-synthase
CT: Computed tomography
DHFR: Dihydrofolate reductase
ECOG-PS: Eastern Cooperative Oncology Group performance status
FA: Folic acid
FN: Febrile neutropenia
FPGS: Folylpoly-γ-glutamate synthase
G: Grade
GGH: γ-Glutamyl hydrolase
Hcy: Homocysteine
HIBCH: 3-Hydroxyisobutyryl-CoA hydrolase
HR: Hazard ratio
LD: Linkage disequilibrium
MPM: Malignant pleural mesothelioma
MTHFR: Methylenetetrahydrofolate reductase
MTR: Methionine synthase
MTRR: Methionine synthase reductase
NLR: Neutrophil to lymphocyte ratio
non-Sq NSCLC: Non-squamous non-small cell lung cancer
OR: Odds ratio
OS: Overall survival
PFS: Progression-free survival
PS: Propensity score
PT: Prothrombin time
RBC: Red blood cell
RBP: Retinol-binding protein
ROC: Receiver operating characteristics
SI: Smoking index
SLC19A1: Folate carrier
SNPs: Single-nucleotide polymorphisms
Tf: Transferrin
TTR: Transthyretin
TYMS: Thymidylate synthase
