# Supplementary material for: Correlation of toxicities and efficacies of pemetrexed with clinical factors and single-nucleotide polymorphisms: a prospective observational study
Source: BMC Cancer. 2023 Aug 26;23:800. doi: 10.1186/s12885-023-11257-8 (PMC10464354; doi:10.1186/s12885-023-11257-8)
Supplement: Supplementary file 2 — Additional file 2: Supplementary Table S1. All types of adverse events during the 1st cycle of pemetrexed-containing chemotherapy. Supplementary Table S2. Risk factors for grades 3 to 4 hematological adverse events at the 1st cycle by logistic regression analysis (N = 71). Supplementary Table S3. Risk factors for grades 2 to 4 non-hematological adverse events at the 1st cycle by logistic regression analysis (N = 71). Supplementary Table S4. Progression-free survival for non-squamous non-small cell lung cancer by Cox regression analyses (N = 63). Supplementary Table S5. Overall survival for non-squamous non-small cell lung cancer by Cox regression analyses (N = 63). [file 12885_2023_11257_MOESM2_ESM.docx]

**Supplementary Table S1.** **All types of adverse events during the 1st cycle of pemetrexed-containing chemotherapy**

|  | Grade 0 | Grade 1 | Grade 2 | Grade 3 | Grade 4 | Grade 5 | Total |
| --- | --- | --- | --- | --- | --- | --- | --- |
| **Hematologic toxicities** |  |  |  |  |  |  |  |
| **Leukopenia** | 32 | 5 | 21 | 12 | 1 | 0 | 71 |
| **Neutropenia** | 27 | 4 | 18 | 15 | 7 | 0 | 71 |
| **Anemia** | 21 | 30 | 13 | 7 | 0 | 0 | 71 |
| **Thrombocytopenia** | 16 | 36 | 9 | 6 | 4 | 0 | 71 |
| **Non-hematologic toxicities** |  |  |  |  |  |  |  |
| AST (aspartate aminotransferase ) | 37 | 26 | 5 | 2 | 1 | 0 | 71 |
| **ALT (alanine aminotransferase** ) | 19 | 38 | 6 | 8 | 0 | 0 | 71 |
| Total bilirubin | 68 | 3 | 0 | 0 | 0 | 0 | 71 |
| Serum creatinine | 53 | 17 | 1 | 0 | 0 | 0 | 71 |
| Fatigue | 39 | 26 | 6 | 0 | 0 | 0 | 71 |
| **Anorexia** | 17 | 26 | 27 | 1 | 0 | 0 | 71 |
| **Nausea** | 36 | 25 | 10 | 0 | 0 | 0 | 71 |
| Vomiting | 66 | 5 | 0 | 0 | 0 | 0 | 71 |
| **Febrile neutropenia** | 58 | 0 | 0 | 12 | 1 | 0 | 71 |
| Pneumonitis | 71 | 0 | 0 | 0 | 0 | 0 | 71 |
| Proteinuria | 63 | 5 | 3 | 0 | 0 | 0 | 71 |
| Nasal bleeding | 68 | 2 | 1 | 0 | 0 | 0 | 71 |
| Skin disorders | 56 | 6 | 8 | 1 | 0 | 0 | 71 |
| fever | 65 | 5 | 1 | 0 | 0 | 0 | 71 |
| Hypertension | 65 | 1 | 5 | 0 | 0 | 0 | 71 |
| Infection | 65 | 0 | 1 | 5 | 0 | 0 | 71 |
| Constipation | 68 | 1 | 2 | 0 | 0 | 0 | 71 |
| Other (diarrhea, Intestinal perforation) | 69 | 0 | 1 | 0 | 1 | 0 | 71 |

CTCAE ver. 4.0, common terminology criteria for adverse events. Bold items mean adverse events frequency ≥14% in Grade 3 to 4 hematological toxicities except for anemia and febrile neutropenia or grade 2 to 4 anemia and non-hematological toxicities except for febrile neutropenia.

**Supplementary Table S2.** **Risk factors for grades 3 to 4 hematological adverse events at the 1st cycle by logistic regression analysis (N = 71)**

**Table S2(a). Crude risk factors**

| **Univariate** | | | | | | **Multivariate** | | | | | |
| --- | --- | --- | --- | --- | --- | --- | --- | --- | --- | --- | --- |
| **Variables** | **OR** | **95% CI** | | | ***P*-value** | **Variables** | **OR** | **95% CI** | | | ***P*-value** |
| **WBC count before pemetrexed (<6120 vs 6120 /µL≤)** | 0.133 | 0.044 | – | 0.403 | **<0.001** | **WBC count before pemetrexed (<6120 vs 6120 /µL≤)** | **0.105** | **0.024** | **–** | **0.457** | **0.003** |
| **Neutrophil count before pemetrexed (≤4126 vs 4127 /µL≤)** | 0.181 | 0.063 | – | 0.524 | **0.002** |  | NI |  |  |  |  |
| **Reduced dose chemotherapy(+ vs -)** | 0.146 | 0.035 | – | 0.605 | **0.008** | **Reduced dose chemotherapy (+ vs -)** | **0.13** | **0.019** | **–** | **0.886** | **0.037** |
| **Hemoglobin before pemetrexed (7.9 → 17.4 g/dL)** | 0.697 | 0.505 | – | 0.963 | **0.029** | Hemoglobin before pemetrexed (7.9 → 17.4 g/dL) | 0.687 | 0.434 | – | 1.086 | 0.108 |
| ***MTHFR a* [677 C>T] (CT+TT vs CC)** | 0.313 | 0.106 | – | 0.924 | **0.036** | ***MTHFR a* [677 C>T] (CT+TT vs CC)** | **0.189** | **0.042** | **–** | **0.842** | **0.029** |
| Vitamin B12 before premedication (<486 vs 486 pg/mL ≤) | 0.407 | 0.152 | – | 1.091 | 0.074 | Vitamin B12 before premedication (<486 vs 486 pg/mL ≤) | 0.305 | 0.077 | – | 1.215 | 0.092 |
| Folic acid before premedication (<6.9 vs 6.9 ng/mL≤) | 0.407 | 0.152 | – | 1.091 | 0.074 | Folic acid before premedication (<6.9 vs 6.9 ng/mL≤) | 0.285 | 0.069 | – | 1.171 | 0.082 |

**Table S2(b). Propensity score-adjusted risk factors**

| **Univariate** | | | | | | **Multivariate** | | | | | | |
| --- | --- | --- | --- | --- | --- | --- | --- | --- | --- | --- | --- | --- |
| **Variables** | **OR** | **95% CI** | | | ***P*-value** | **Variables** | **OR** | **95% CI** | | | ***P*-value** |  |
| **WBC count before pemetrexed (<6120 vs 6120 /µL≤)** | 0.131 | 0.043 | – | 0.4 | **<0.001** | **WBC count before pemetrexed (<6120 vs 6120 /µL≤)** | **0.064** | **0.012** | **–** | **0.346** | **0.001** |  |
| **Neutrophil count before pemetrexed (≤4126 vs 4127 /µL≤)** | 0.167 | 0.056 | – | 0.5 | **0.001** |  | NI |  |  |  |  |  |
| **Reduced dose chemotherapy (+ vs -)** | 0.144 | 0.034 | – | 0.598 | **0.008** | **Reduced dose chemotherapy (+ vs -)** | **0.093** | **0.01** | **–** | **0.905** | **0.041** |  |
| **Hemoglobin before pemetrexed (7.9 → 17.4 g/dL)** | 0.698 | 0.505 | – | 0.964 | **0.029** | Hemoglobin before pemetrexed (7.9 → 17.4 g/dL) | 0.602 | 0.347 | – | 1.044 | 0.071 |  |
| ***MTHFR a* [677 C>T] (CT+TT vs CC)** | 0.281 | 0.089 | – | 0.893 | **0.031** | ***MTHFR a* [677 C>T] (CT+TT vs CC)** | **0.131** | **0.019** | **–** | **0.922** | **0.041** |  |
| Folic acid before premedication (<6.9 vs 6.9 ng/mL≤) | 0.388 | 0.139 | – | 1.085 | 0.071 |  | NI |  |  |  |  |  |
| Vitamin B12 before premedication (<486 vs 486 pg/mL ≤) | 0.408 | 0.152 | – | 1.094 | 0.075 | **Vitamin B12 before premedication (<486 vs 486 pg/mL≤)** | **0.134** | **0.025** | **–** | **0.725** | **0.02** |  |
| Folic acid before pemetrexed (<15.8 vs 15.8 ng/mL≤) | 0.393 | 0.134 | – | 1.154 | 0.089 | **Folic acid before pemetrexed (<15.8 vs 15.8 ng/mL≤)** | **0.15** | **0.025** | **–** | **0.893** | **0.037** |  |
| Duration of premedication (42 → 6 days) | 0.94 | 0.873 | – | 1.012 | 0.099 |  | NI |  |  |  |  |  |

Abbreviations: before pemetrexed, at the start of pemetrexed treatment**;** CI, confidence interval; *MTHFR*, methylenetetrahydrofolate reductase; NI, not included in the final multivariate logistic regression model; OR, Odds ratio; WBC, white blood cell. Variables with a *P***-**value < 0.10 on univariate analysis were entered into multivariate logistical analysis by a simultaneous method. Bold items mean *P*-value < 0.05.

**Supplementary Table S3.** **Risk factors for grades 2 to 4 non-hematological adverse events at the 1st cycle by logistic regression analysis (N = 71)**

**Table S3(a). Crude risk factors**

| **Univariate** | | | | | | **Multivariate** | | | | | |
| --- | --- | --- | --- | --- | --- | --- | --- | --- | --- | --- | --- |
| **Variables** | **OR** | **95% CI** | | | ***P*-value** | **Variables** | **OR** | **95% CI** | | | ***P*-value** |
| ***MTHFR a* [677 C>T] (CC+TT vs CT)** | 0.186 | 0.059 | – | 0.589 | **0.004** | ***MTHFR a* [677 C>T] (CC+TT vs CT)** | **0.108** | **0.018** | **–** | **0.642** | **0.014** |
| **RBP before pemetrexed (<2.95 vs 2.95 mg/dL≤)** | 0.184 | 0.052 | – | 0.647 | **0.008** | **RBP before pemetrexed (<2.95 vs 2.95 mg/dL≤)** | **0.054** | **0.006** | **–** | **0.474** | **0.008** |
| ***MTHFR a* [677 C>T] (CC vs CT+TT)** | 0.164 | 0.043 | – | 0.628 | **0.008** |  | NI |  |  |  |  |
| **Vitamin B12 before pemetrexed (<664 vs 664 pg/mL≤)** | 0.254 | 0.084 | – | 0.768 | **0.015** |  | NI |  |  |  |  |
| **Vitamin B12 before premedication (<486 vs 486 pg/mL≤)** | 0.259 | 0.086 | – | 0.775 | **0.016** | **Vitamin B12 before premedication (<486 vs 486 pg/mL≤)** | **0.026** | **0.002** | **–** | **0.316** | **0.004** |
| **Serum urea nitrogen before pemetrexed (<14.1 vs 14.1 mg/dL≤)** | 0.259 | 0.086 | – | 0.775 | **0.016** |  | NI |  |  |  |  |
| **RBC count before pemetrexed (<400 vs 400x10^4^ /µL≤)** | 0.281 | 0.094 | – | 0.841 | **0.023** | **RBC count before pemetrexed (<400 vs 400x10^4^ /µL≤)** | **0.162** | **0.027** | **–** | **0.97** | **0.046** |
| **Transthyretin before pemetrexed (<21.5 vs 21.5 mg/dL≤)** | 0.271 | 0.083 | – | 0.887 | **0.031** |  | NI |  |  |  |  |
| **Homocysteine before premedication (11.8 ≤ vs <11.8 nmol/mL)** | 0.297 | 0.096 | – | 0.918 | **0.035** |  | NI |  |  |  |  |
| **Driver mutation (+ vs -)** | 0.273 | 0.08 | – | 0.924 | **0.037** |  | NI |  |  |  |  |
| **Body mass index before pemetrexed (≤21.7 vs 21.8 kg/m^2^≤)** | 0.322 | 0.111 | – | 0.931 | **0.037** |  | NI |  |  |  |  |
| Folic acid before premedication (<6.9 vs 6.9 ng/mL≤) | 0.35 | 0.121 | – | 1.011 | 0.052 | Folic acid before premedication (<6.9 vs 6.9 ng/mL≤) | 0.151 | 0.019 | – | 1.202 | 0.074 |
| *DHFR* [680 C>A] (AC+CC vs AA) | 0.361 | 0.126 | – | 1.031 | 0.057 |  | NI |  |  |  |  |
| Platelet count before pemetrexed (23.8≤ vs <23.8x10^4^ /µL) | 0.394 | 0.139 | – | 1.114 | 0.079 |  | NI |  |  |  |  |
| Hemoglobin before pemetrexed (7.9 → 17.4 g/dL) | 0.759 | 0.556 | – | 1.035 | 0.081 |  | NI |  |  |  |  |
| *SLC19A1 a* [IVS2 (4935) G>A] (AA+GG vs AG) | 0.361 | 0.115 | – | 1.134 | 0.081 |  | NI |  |  |  |  |
| *SLC19A1 a* [IVS2 (4935) G>A] (GG vs AA+AG) | 0.165 | 0.02 | – | 1.364 | 0.094 |  | NI |  |  |  |  |

**Table S3(b). Propensity score-adjusted risk factors**

| **Univariate** | | | | | | **Multivariate** | | | | | | |
| --- | --- | --- | --- | --- | --- | --- | --- | --- | --- | --- | --- | --- |
| **Variables** | **OR** | **95% CI** | | | ***P*-value** | **Variables** | **OR** | **95% CI** | | | ***P*-value** |  |
| ***MTHFR a* [677 C>T] (CC+TT vs CT)** | 0.198 | 0.061 | – | 0.644 | **0.007** | ***MTHFR a* [677 C>T] (CC+TT vs CT)** | **0.071** | **0.009** | – | **0.536** | **0.010** |  |
| **RBP before pemetrexed (<2.95 vs 2.95 mg/dL≤)** | 0.184 | 0.052 | – | 0.650 | **0.009** |  | NI |  |  |  |  |  |
| **Homocysteine before premedication (11.8 ≤ vs <11.8 nmol/mL)** | 0.200 | 0.057 | – | 0.705 | **0.012** | **Homocysteine before premedication (11.8≤ vs <11.8 nmol/mL)** | **0.029** | **0.003** | – | **0.302** | **0.003** |  |
| **Vitamin B12 before premedication (<486 vs 486 pg/mL≤)** | 0.238 | 0.077 | – | 0.735 | **0.013** |  | NI |  |  |  |  |  |
| ***MTHFR a* [677 C>T] (CC vs CT+TT)** | 0.175 | 0.044 | – | 0.695 | **0.013** |  | NI |  |  |  |  |  |
| **Vitamin B12 before pemetrexed (<664 vs 664 pg/mL≤)** | 0.252 | 0.082 | – | 0.773 | **0.016** |  | NI |  |  |  |  |  |
| **Folic acid before premedication (<6.9 vs 6.9 ng/mL≤)** | 0.259 | 0.082 | – | 0.817 | **0.021** |  | NI |  |  |  |  |  |
| **RBC count before pemetrexed (<400 vs 400x10^4^ /µL≤)** | 0.268 | 0.088 | – | 0.816 | **0.021** |  | NI |  |  |  |  |  |
| **Transthyretin before pemetrexed (<21.5 vs 21.5 mg/dL≤)** | 0.260 | 0.078 | – | 0.862 | **0.028** | **Transthyretin before pemetrexed (<21.5 vs 21.5 mg/dL≤)** | **0.056** | **0.006** | – | **0.500** | **0.010** |  |
| **Serum urea nitrogen before pemetrexed (<14.1 vs 14.1 mg/dL≤)** | 0.269 | 0.082 | – | 0.889 | **0.031** |  | NI |  |  |  |  |  |
| *DHFR* [680 C>A] (AC+CC vs AA) | 0.350 | 0.121 | – | 1.015 | 0.053 |  | NI |  |  |  |  |  |
| Driver mutation (+ vs -) | 0.295 | 0.085 | – | 1.019 | 0.054 |  | NI |  |  |  |  |  |
| Body mass index before pemetrexed (≤21.7 vs 21.8 kg/m^2^≤) | 0.351 | 0.118 | – | 1.041 | 0.059 |  | NI |  |  |  |  |  |
| Performance status before pemetrexed (2<1<0) | 0.403 | 0.153 | – | 1.058 | 0.065 |  | NI |  |  |  |  |  |
| C-reactive protein before pemetrexed (24.47 → 0.02 mg/dL) | 0.731 | 0.521 | – | 1.025 | 0.069 |  | NI |  |  |  |  |  |
| Hemoglobin before pemetrexed (7.9 → 17.4 g/dL) | 0.755 | 0.555 | – | 1.028 | 0.074 |  | NI |  |  |  |  |  |
| *SLC19A1 a* [IVS2 (4935) G>A] (GG vs AA+AG) | 0.145 | 0.017 | – | 1.223 | 0.076 |  | NI |  |  |  |  |  |
| *SLC19A1 a* [IVS2 (4935) G>A] (AA+GG vs AG) | 0.363 | 0.114 | – | 1.152 | 0.085 | ***SLC19A1 a* [IVS2 (4935) G>A] (AA+GG vs AG)** | **0.055** | **0.005** | – | **0.576** | **0.016** |  |
| Platelet count before pemetrexed (23.8≤ vs <23.8x10^4^ /µL) | 0.403 | 0.141 | – | 1.148 | 0.089 |  | NI |  |  |  |  |  |
| Saturation of peripheral oxygen before pemetrexed (93 → 99 %) | 0.641 | 0.381 | – | 1.081 | 0.095 |  | NI |  |  |  |  |  |

Abbreviations: before pemetrexed, at the start of pemetrexed treatment; CI, confidence interval; *DHFR*, Dihydrofolate reductase; *MTHFR*, methylenetetrahydrofolate reductase; NI, not included in the final multivariate logistic regression model; OR, Odds ratio; RBC, red blood cell; RBP, retinol-binding protein; SLC19A1, Folate carrier.

Variables with a *P***-**value < 0.10 on univariate analysis were entered into multivariate logistical analysis by a simultaneous method. Bold items mean *P*-value < 0.05.

**Supplementary Table S4.** **Progression-free survival for non-squamous non-small cell lung cancer by Cox regression analyses (N = 63)**

**Table S4(a). Crude Progression-free survival**

| **Univariate** | | | | | | **Multivariate** | | | | | |
| --- | --- | --- | --- | --- | --- | --- | --- | --- | --- | --- | --- |
| **Variables** | **HR** | **95% CI** | | | ***P*-value** | **Variables** | **HR** | **95% CI** | | | ***P*-value** |
| **Folic acid before pemetrexed (<15.75 vs 15.75 ng/mL≤)** | 0.349 | 0.200 | – | 0.609 | **<0.001** |  | NI |  |  |  |  |
| **C-reactive protein before pemetrexed (1.27≤ vs <1.27 mg/dL)** | 0.281 | 0.149 | – | 0.531 | **<0.001** |  | NI |  |  |  |  |
| **Transferrin before pemetrexed (<203.9 vs 203.9 mg/dL≤)** | 0.219 | 0.108 | – | 0.444 | **<0.001** |  | NI |  |  |  |  |
| **Transthyretin before pemetrexed (<20.2 vs 20.2 mg/dL≤)** | 0.373 | 0.211 | – | 0.659 | **<0.001** | Transthyretin before pemetrexed (<20.2 vs 20.2 mg/dL≤) | 0.760 | 0.374 | – | 1.542 | 0.447 |
| **Retinol binding protein before pemetrexed (0.8 → 6.3 mg/dL)** | 0.613 | 0.466 | – | 0.807 | **<0.001** |  | NI |  |  |  |  |
| **Regimen (Pemetrexed monotherapy vs Platinum+Pemetrexed)** | 0.325 | 0.163 | – | 0.649 | **0.001** | **Regimen (Pemetrexed monotherapy vs Platinum+Pemetrexed)** | **0.219** | **0.090** | **–** | **0.537** | **0.001** |
| **Bevacizumab combination (- vs +)** | 0.398 | 0.219 | – | 0.723 | **0.002** | **Bevacizumab combination (- vs +)** | **0.471** | **0.230** | **–** | **0.962** | **0.039** |
| **Albumin before pemetrexed (2.2 → 4.9 g/dL)** | 0.509 | 0.328 | – | 0.790 | **0.003** |  | NI |  |  |  |  |
| **Hemoglobin before pemetrexed (<11.35 vs 11.35 g/dL≤)** | 0.446 | 0.248 | – | 0.804 | **0.007** |  | NI |  |  |  |  |
| **PLR before pemetrexed (277.9≤ vs <277.9)** | 0.465 | 0.263 | – | 0.822 | **0.008** |  | NI |  |  |  |  |
| **Vitamin B12 before pemetrexed (1136≤ vs <1136 pg/mL)** | 0.425 | 0.222 | – | 0.812 | **0.010** | **Vitamin B12 before pemetrexed (1136≤ vs <1136 pg/mL)** | **0.404** | **0.191** | – | **0.852** | **0.017** |
| **Total Bilirubin before pemetrexed (<0.45 vs 0.45 mg/dL≤)** | 0.506 | 0.296 | – | 0.865 | **0.013** |  | NI |  |  |  |  |
| **Folic acid before premedication (<9.3 vs 9.3 ng/mL≤)** | 0.479 | 0.266 | – | 0.863 | **0.014** | **Folic acid before premedication (<9.3 vs 9.3 ng/mL≤)** | **0.161** | **0.072** | **–** | **0.359** | **<0.001** |
| ***BHMT* [742 G>A] (GG vs AG+AA)** | 0.534 | 0.313 | – | 0.914 | **0.022** | ***BHMT* [742 G>A] (GG vs AG+AA)** | **0.223** | **0.105** | **–** | **0.474** | **<0.001** |
| **ALT before pemetrexed (<13.5 vs 13.5 U/L≤)** | 0.540 | 0.315 | – | 0.923 | **0.024** |  | NI |  |  |  |  |
| **Fibrinogen before pemetrexed (391≤ vs <391 mg/dL)** | 0.554 | 0.330 | – | 0.928 | **0.025** |  | NI |  |  |  |  |
| ***TYMS* [VNTR] (others vs 2R/3R)** | 0.545 | 0.317 | – | 0.938 | **0.028** |  | NI |  |  |  |  |
| **Body mass index before pemetrexed (<21.85 vs 21.85 kg/m^2^≤)** | 0.561 | 0.334 | – | 0.943 | **0.029** | Body mass index before pemetrexed (<21.85 vs 21.85 kg/m^2^≤) | 0.569 | 0.298 | – | 1.086 | 0.087 |
| **Performance status before pemetrexed (2<1<0)** | 0.598 | 0.367 | – | 0.974 | **0.039** |  | NI |  |  |  |  |
| **Duration of premedication (<8.5 vs 8.5 days≤)** | 0.577 | 0.341 | – | 0.976 | **0.040** |  | NI |  |  |  |  |
| ***TYMS* [VNTR] (3R vs 2R)** | 0.578 | 0.339 | – | 0.988 | **0.045** |  | NI |  |  |  |  |
| *GGH* [IVS5 (1042) T>C] (CT vs CC+TT) | 0.603 | 0.356 | – | 1.020 | 0.059 |  | NI |  |  |  |  |
| *GGH* [IVS5 (1042) T>C] (CT+TT vs CC) | 0.588 | 0.338 | – | 1.022 | 0.060 |  | NI |  |  |  |  |
| NLR before pemetrexed (3.51≤ vs <3.51) | 0.629 | 0.376 | – | 1.053 | 0.078 | **NLR before pemetrexed (3.51≤ vs <3.51)** | **0.406** | **0.199** | **–** | **0.830** | **0.013** |
| *TYMS* [VNTR] (3R/3R vs others) | 0.622 | 0.366 | – | 1.057 | 0.079 |  | NI |  |  |  |  |
| Total protein before pemetrexed (<7.05 vs 7.05 g/dL≤) | 0.606 | 0.339 | – | 1.083 | 0.091 | Total protein before pemetrexed (7.05≤ vs <7.05 g/dL) | 0.833 | 0.426 | – | 1.626 | 0.592 |
| INR before pemetrexed (1.96 → 0.79) | 0.405 | 0.140 | – | 1.174 | 0.096 |  | NI |  |  |  |  |
| *BHMT* [742 G>A] (GG+AA vs AG) | 0.623 | 0.357 | – | 1.087 | 0.096 |  | NI |  |  |  |  |

**Table S4(b). Propensity score-adjusted progression-free survival**

| **Univariate** | | | | | | **Multivariate** | | | | | |
| --- | --- | --- | --- | --- | --- | --- | --- | --- | --- | --- | --- |
| **Variables** | **HR** | **95% CI** | | | ***P*-value** | **Variables** | **HR** | **95% CI** | | | ***P*-value** |
| **C-reactive protein before pemetrexed (1.27≤ vs <1.27 mg/dL)** | 0.272 | 0.142 | – | 0.519 | **<0.001** |  | NI |  |  |  |  |
| **Transferrin before pemetrexed (<203.9 vs 203.9 mg/dL≤)** | 0.241 | 0.117 | – | 0.495 | **<0.001** |  | NI |  |  |  |  |
| **Transthyretin before pemetrexed (<20.2 vs 20.2 mg/dL≤)** | 0.289 | 0.155 | – | 0.54 | **<0.001** | Transthyretin before pemetrexed (<20.2 vs 20.2 mg/dL≤) | 0.529 | 0.260 | – | 1.079 | 0.08 |
| **Retinol binding protein before pemetrexed (0.8 → 6.3 mg/dL)** | 0.572 | 0.427 | – | 0.766 | **<0.001** |  | NI |  |  |  |  |
| **Regimen (Pemetrexed monotherapy vs Platinum+Pemetrexed)** | 0.318 | 0.159 | – | 0.638 | **0.001** | **Regimen (Pemetrexed monotherapy vs Platinum+Pemetrexed)** | **0.266** | **0.109** | **–** | **0.651** | **0.004** |
| **Folic acid before pemetrexed (<15.75 vs 15.75 ng/mL≤)** | 0.380 | 0.212 | – | 0.681 | **0.001** |  | NI |  |  |  |  |
| **Albumin before pemetrexed (2.2 → 4.9 g/dL)** | 0.507 | 0.325 | – | 0.791 | **0.003** |  | NI |  |  |  |  |
| ***BHMT* [742 G>A] (GG vs AG+AA)** | 0.485 | 0.282 | – | 0.835 | **0.009** | ***BHMT* [742 G>A] (GG vs AG+AA)** | **0.237** | **0.107** | **–** | **0.527** | **<0.001** |
| **Folic acid before premedication (<9.3 vs 9.3 ng/mL≤)** | 0.497 | 0.275 | – | 0.898 | **0.02** | **Folic acid before premedication (<9.3 vs 9.3 ng/mL≤)** | **0.135** | **0.058** | **–** | **0.316** | **<0.001** |
| **Hemoglobin before pemetrexed (7.9 → 17.4 g/dL)** | 0.826 | 0.703 | – | 0.971 | **0.02** |  | NI |  |  |  |  |
| **PLR before pemetrexed (277.9≤ vs <277.9)** | 0.509 | 0.287 | – | 0.903 | **0.021** |  | NI |  |  |  |  |
| **Bevacizumab combination (- vs +)** | 0.402 | 0.184 | – | 0.881 | **0.023** | **Bevacizumab combination (- vs +)** | **0.365** | **0.137** | **–** | **0.972** | **0.044** |
| **Vitamin B12 before pemetrexed (1136≤ vs <1136 pg/mL)** | 0.466 | 0.242 | – | 0.901 | **0.023** | **Vitamin B12 before pemetrexed (1136≤ vs <1136 pg/mL)** | **0.352** | **0.169** | **–** | **0.734** | **0.005** |
| **Total protein before pemetrexed (<7.05 vs 7.05 g/dL≤)** | 0.500 | 0.272 | – | 0.92 | **0.026** | Total protein before pemetrexed (<7.05 vs 7.05 g/dL≤) | 0.812 | 0.403 | – | 1.639 | 0.562 |
| **ALT before pemetrexed (<13.5 vs 13.5 U/L≤)** | 0.558 | 0.326 | – | 0.957 | **0.034** |  | NI |  |  |  |  |
| **Fibrinogen before pemetrexed (391≤ vs <391 mg/dL)** | 0.571 | 0.341 | – | 0.958 | **0.034** |  | NI |  |  |  |  |
| **Performance status before pemetrexed (2<1<0)** | 0.599 | 0.370 | – | 0.971 | **0.037** |  | NI |  |  |  |  |
| **Body mass index before pemetrexed (<21.85 vs 21.85 kg/m^2^≤)** | 0.581 | 0.347 | – | 0.975 | **0.04** |  | NI |  |  |  |  |
| ***DHFR* [680 C>A] (CC vs AC+AA)** | 0.452 | 0.211 | – | 0.969 | **0.041** | ***DHFR* [680 C>A] (CC vs AC+AA)** | **0.194** | **0.068** | **–** | **0.554** | **0.002** |
| Total Bilirubin before pemetrexed (<0.45 vs 0.45 mg/dL≤) | 0.581 | 0.332 | – | 1.016 | 0.057 |  | NI |  |  |  |  |
| *BHMT* [742 G>A] (GG+AA vs AG) | 0.579 | 0.330 | – | 1.018 | 0.058 |  | NI |  |  |  |  |
| *GGH* [IVS5 (1042) T>C] (CT+TT vs CC) | 0.587 | 0.337 | – | 1.019 | 0.059 | *GGH* [IVS5 (1042) T>C] (CT+TT vs CC) | 0.510 | 0.258 | – | 1.008 | 0.053 |
| *TYMS* [VNTR] (others vs 2R/3R) | 0.596 | 0.342 | – | 1.039 | 0.068 |  | NI |  |  |  |  |
| *GGH* [IVS5 (1042) T>C] (CT vs CC+TT) | 0.635 | 0.376 | – | 1.074 | 0.091 |  | NI |  |  |  |  |

Abbreviations: ALT, alanine aminotransferase; before pemetrexed, at the start of pemetrexed treatment; *BHMT*, betaine-homocysteine methyltransferase; CI, confidence interval; *DHFR*, dihydrofolate reductase; GGH, γ-glutamyl hydrolase; HR, Hazard ratio; INR, the international normalized ratio of prothrombin time; NI, not included in the final multivariate Cox regression model; NLR, neutrophil/lymphocyte ratio; PLR, platelet/lymphocyte ratio; *TYMS*, thymidylate synthase. Variables with a *P*-value < 0.10 on univariate analysis were entered into multivariate Cox analysis by a simultaneous method. Bold items mean *P*-value < 0.05.

**Supplementary Table S5.** **Overall survival for non-squamous non-small cell lung cancer by Cox regression analyses (N = 63)**

**Table S5(a). Crude overall survival**

| **Univariate** | | | | | | **Multivariate** | | | | | |
| --- | --- | --- | --- | --- | --- | --- | --- | --- | --- | --- | --- |
| **Variables** | **HR** | **95% CI *P*-value** | | | | **Variables** | **HR** | **95% CI** | | | ***P*-value** |
| **Performance status before pemetrexed (2>1> 0)** | 0.277 | 0.154 | – | 0.498 | **<0.001** | **Performance status before pemetrexed (2>1>0)** | **0.233** | **0.120** | **–** | **0.452** | **<0.001** |
| **Body mass index before pemetrexed (<20.66 vs 20.66 kg/m^2^≤)** | 0.310 | 0.162 | – | 0.593 | **<0.001** | **Body mass index before pemetrexed (<20.66 vs 20.66 kg/m^2^≤)** | **0.146** | **0.063** | **–** | **0.338** | **<0.001** |
| **Folic acid before premedication (<5.55 vs 5.55 ng/mL≤)** | 0.311 | 0.159 | – | 0.606 | **<0.001** | **Folic acid before premedication (<5.55 vs 5.55 ng/mL≤)** | **0.213** | **0.093** | **–** | **0.487** | **<0.001** |
| **C-reactive protein before pemetrexed (1.435≤ vs <1.435 mg/dL)** | 0.137 | 0.062 | – | 0.306 | **<0.001** |  | NI |  |  |  |  |
| **RBP before pemetrexed (0.8 → 6.3 mg/dL)** | 0.589 | 0.426 | – | 0.815 | **0.001** | **RBP before pemetrexed (0.8 → 6.3 mg/dL)** | **0.547** | **0.371** | **–** | **0.805** | **0.002** |
| **Fibrinogen before pemetrexed (484.5≤ vs <484.5 mg/dL)** | 0.350 | 0.168 | – | 0.727 | **0.005** |  | NI |  |  |  |  |
| **ALT before pemetrexed (<15 vs 15 U/L≤)** | 0.399 | 0.206 | – | 0.774 | **0.007** |  | NI |  |  |  |  |
| ***MTRR* [66 A >G] (GG vs AG+AA)** | 0.222 | 0.075 | – | 0.658 | **0.007** |  | NI |  |  |  |  |
| **Transthyretin before pemetrexed (<19.5 vs 19.5 mg/dL≤)** | 0.443 | 0.234 | – | 0.841 | **0.013** |  | NI |  |  |  |  |
| **eGFR before pemetrexed (76≤ vs <76 mL/min)** | 0.473 | 0.255 | – | 0.875 | **0.017** |  | NI |  |  |  |  |
| **Hemoglobin before pemetrexed (7.9 → 17.4 g/dL)** | 0.787 | 0.644 | – | 0.961 | **0.019** |  | NI |  |  |  |  |
| **Total bilirubin before pemetrexed (<0.45 vs 0.45 mg/dL≤)** | 0.477 | 0.255 | – | 0.891 | **0.02** |  | NI |  |  |  |  |
| **Smoking index (22.5≤ vs <22.5)** | 0.490 | 0.257 | – | 0.937 | **0.031** | **Smoking index (22.5≤ vs <22.5)** | **0.207** | **0.094** | **–** | **0.455** | **<0.001** |
| ***BHMT* [742 G>A] (AG+GG vs AA)** | 0.112 | 0.015 | – | 0.826 | **0.032** | *BHMT* [742 G>A] (AG+GG vs AA) | 0.275 | 0.035 | – | 2.151 | 0.219 |
| **Gender (male vs female)** | 0.447 | 0.213 | – | 0.939 | **0.033** |  | NI |  |  |  |  |
| **Urologic and renal comorbidities (+ vs -)** | 0.263 | 0.077 | – | 0.895 | **0.033** |  | NI |  |  |  |  |
| **Driver mutation (- vs +)** | 0.505 | 0.263 | – | 0.969 | **0.04** |  | NI |  |  |  |  |
| **RBC count before pemetrexed (<390.5 vs 390.5x10^4^/μL≤)** | 0.532 | 0.290 | – | 0.977 | **0.042** |  | NI |  |  |  |  |
| ***MTHFR* [1298 A>C] (CC vs AC+AA)** | 0.223 | 0.051 | – | 0.980 | **0.047** |  | NI |  |  |  |  |
| Albumin before pemetrexed (<3.05 vs 3.05 g/dL≤) | 0.517 | 0.263 | – | 1.015 | 0.055 |  | NI |  |  |  |  |
| INR before pemetrexed (1.02≤ vs <1.02) | 0.567 | 0.312 | – | 1.028 | 0.062 |  | NI |  |  |  |  |
| *SLC19A1* [IVS2 (4935) G>A] (GG vs AG+AA) | 0.483 | 0.222 | – | 1.046 | 0.065 |  | NI |  |  |  |  |
| *HIBCH* [c.2T>C p. Met1] (TT vs CT) | 0.535 | 0.275 | – | 1.043 | 0.066 |  | NI |  |  |  |  |
| Duration of premedication (14.5≤ vs <14.5 days) | 0.529 | 0.268 | – | 1.045 | 0.067 |  | NI |  |  |  |  |
| Transferrin before pemetrexed (<245 vs 245 mg/dL≤) | 0.559 | 0.297 | – | 1.055 | 0.073 |  | NI |  |  |  |  |
| Bevacizumab combination (- vs +) | 0.534 | 0.268 | – | 1.064 | 0.074 |  | NI |  |  |  |  |
| *MTRR* [66 A >G] (AA+GG vs AG) | 0.572 | 0.309 | – | 1.059 | 0.075 | ***MTRR* [66 A >G] (AA+GG vs AG)** | **0.344** | **0.174** | **–** | **0.684** | **0.002** |
| AST before pemetrexed (<20.5 vs 20.5 U/L≤) | 0.595 | 0.323 | – | 1.097 | 0.096 |  | NI |  |  |  |  |

**Table S5(b). Propensity score-adjusted overall survival**

| **Univariate** | | | | | | **Multivariate** | | | | | |
| --- | --- | --- | --- | --- | --- | --- | --- | --- | --- | --- | --- |
| **Variables** | **HR** | **95% CI** | | | ***P*-value** | **Variables** | **HR** | **95% CI** | | | ***P*-value** |
| **Performance status before pemetrexed (2>1>0)** | 0.269 | 0.151 | – | 0.482 | **<0.001** | **Performance status before pemetrexed (2>1>0)** | **0.223** | **0.111** | **–** | **0.394** | **<0.001** |
| **Body mass index before pemetrexed (<20.66 vs 20.66 kg/m^2^≤)** | 0.263 | 0.132 | – | 0.525 | **<0.001** | **Body mass index before pemetrexed (<20.66 vs 20.66 kg/m^2^≤)** | **0.160** | **0.068** | **–** | **0.374** | **<0.001** |
| **Folic acid before premedication (<5.55 vs 5.55 ng/mL≤)** | 0.300 | 0.147 | – | 0.609 | **<0.001** | **Folic acid before premedication (<5.55 vs 5.55 ng/mL≤)** | **0.182** | **0.072** | **–** | **0.462** | **<0.001** |
| **C-reactive protein before pemetrexed (1.435≤ vs <1.435 mg/dL)** | 0.135 | 0.060 | – | 0.303 | **<0.001** |  | NI |  |  |  |  |
| **RBP before pemetrexed (0.8 → 6.3 mg/dL)** | 0.561 | 0.399 | – | 0.789 | **<0.001** | **RBP before pemetrexed (0.8 → 6.3 mg/dL)** | **0.559** | **0.380** | **–** | **0.821** | **0.003** |
| **INR before pemetrexed (1.02≤ vs <1.02)** | 0.150 | 0.042 | – | 0.536 | **0.004** |  | NI |  |  |  |  |
| **Fibrinogen before pemetrexed (484.5≤ vs <484.5 mg/dL)** | 0.340 | 0.162 | – | 0.710 | **0.004** |  | NI |  |  |  |  |
| **Transthyretin before pemetrexed (<19.5 vs 19.5 mg/dL≤)** | 0.389 | 0.198 | – | 0.765 | **0.006** |  | NI |  |  |  |  |
| **ALT before pemetrexed (<15 vs 15 U/L≤)** | 0.404 | 0.207 | – | 0.787 | **0.008** |  | NI |  |  |  |  |
| ***MTRR* [66 A >G] (GG vs AG+AA)** | 0.228 | 0.075 | – | 0.692 | **0.009** |  | NI |  |  |  |  |
| **Hemoglobin before pemetrexed (7.9 → 17.4 g/dL)** | 0.391 | 0.187 | – | 0.820 | **0.013** |  | NI |  |  |  |  |
| ***BHMT* [742 G>A] (AG+GG vs AA)** | 0.088 | 0.011 | – | 0.673 | **0.019** | *BHMT* [742 G>A] (AG+GG vs AA) | 0.226 | 0.029 | – | 1.761 | 0.156 |
| **eGFR before pemetrexed (76≤ vs <76mL/min)** | 0.478 | 0.257 | – | 0.889 | **0.020** |  | NI |  |  |  |  |
| **Total bilirubin before pemetrexed (<0.45 vs 0.45 mg/dL≤)** | 0.482 | 0.257 | – | 0.905 | **0.023** |  | NI |  |  |  |  |
| **Gender (male vs female)** | 0.386 | 0.164 | – | 0.904 | **0.028** |  | NI |  |  |  |  |
| **Urologic and renal comorbidities (+ vs -)** | 0.254 | 0.074 | – | 0.870 | **0.029** |  | NI |  |  |  |  |
| **Smoking index (22.5≤ vs <22.5)** | 0.471 | 0.233 | – | 0.950 | **0.035** | **Smoking index (22.5≤ vs <22.5)** | **0.155** | **0.063** | **–** | **0.383** | **<0.001** |
| **RBC count before pemetrexed (<390.5 vs 390.5x 10^4^/μL≤)** | 0.520 | 0.282 | – | 0.957 | **0.036** |  | NI |  |  |  |  |
| **Bevacizumab combination (- vs +)** | 0.365 | 0.142 | – | 0.939 | **0.037** |  | NI |  |  |  |  |
| ***HIBCH* [c.2T>C p. Met1] (TT vs CT)** | 0.480 | 0.240 | – | 0.960 | **0.038** |  | NI |  |  |  |  |
| **Driver mutation (- vs +)** | 0.509 | 0.265 | – | 0.977 | **0.042** |  | NI |  |  |  |  |
| ***MTHFR* [1298 A>C] (CC vs AC+AA)** | 0.226 | 0.051 | – | 0.991 | **0.049** |  | NI |  |  |  |  |
| Albumin before pemetrexed (<3.05 vs 3.05 g/dL≤) | 0.513 | 0.261 | – | 1.010 | 0.053 |  | NI |  |  |  |  |
| Duration of premedication (14.5≤ vs <14.5 days) | 0.525 | 0.266 | – | 1.036 | 0.063 |  | NI |  |  |  |  |
| *SLC19A1* [IVS2 (4935) G>A] (GG vs AG+AA) | 0.490 | 0.225 | – | 1.066 | 0.072 |  | NI |  |  |  |  |
| *MTRR* [66 A >G] (AA+GG vs AG) | 0.577 | 0.311 | – | 1.070 | 0.081 | ***MTRR* [66 A >G] (AA+GG vs AG)** | **0.348** | **0.174** | **–** | **0.697** | **0.003** |
| Neuropsychiatric comorbidity (- vs +) | 0.403 | 0.138 | – | 1.175 | 0.096 | Neuropsychiatric comorbidity (- vs +) | 0.330 | 0.101 | – | 1.076 | 0.066 |
| Reduced dose chemotherapy (+ vs -) | 0.507 | 0.228 | – | 1.127 | 0.096 |  | NI |  |  |  |  |

Abbreviations: ALT, alanine aminotransferase; AST, aspartate aminotransferase; before pemetrexed, at the start of pemetrexed treatment; *BHMT*, betaine-homocysteine methyltransferase; CI, confidence interval; eGFR, estimated glomerular filtration rate; *HIBCH*, 3-Hydroxyisobutyryl-CoA hydrolase; HR, Hazard ratio; INR, the international normalized ratio of prothrombin time; *MTHFR*, methylenetetrahydrofolate reductase; *MTRR*, methionine synthase reductase; NI, not included in the final multivariate Cox regression model; RBC, red blood cell; RBP, retinol-binding protein; *SLC19A1*, folate carrier.

Variables with a *P*-value < 0.10 on univariate analysis were entered into multivariate Cox analysis by a simultaneous method. Bold items mean *P*-value < 0.05.
