# Supplementary material for: Correlation of toxicities and efficacies of pemetrexed with clinical factors and single-nucleotide polymorphisms: a prospective observational study
Source: BMC Cancer. 2023 Aug 26;23:800. doi: 10.1186/s12885-023-11257-8 (PMC10464354; doi:10.1186/s12885-023-11257-8)

**Correlation of toxicities and efficacies of pemetrexed with clinical factors and single-nucleotide polymorphisms: a prospective observational study**

**Supplement Fig.S1: Propensity score-adjusted risk factors for toxicities at the 1st cycle by multivariate logistic regression analysis (N = 71).**

Fig.S1(a) G3 to 4 leucopenia, Fig.S1(b) G3 to 4 neutropenia, Fig.S1(c) G2 to 4 anemia, Fig.S1(d) G3 to 4 thrombocytopenia, Fig.S1(e) G2 to 4 serum ALT elevation, Fig.S1(f) G2 to 4 anorexia, Fig.S1(g) G2 to 4 nausea, Fig.S1(h) G3 to 4 febrile neutropenia.

ALT, alanine aminotransferase; before pemetrexed, at the start of pemetrexed treatment; *DHFR*, dihydrofolate reductase; *FPGS*, folylpoly-γ-glutamate synthase; G, toxic grade; *MTHFR*, methylenetetrahydrofolate reductase; OR, odds ratio; RBC, red blood cell; *SLC19A1*, folate carrier; WBC, white blood cell;95%CI, 95% confidence interval.

Variables with a *P*-value < 0.10 on univariate analysis in Fig.S1(a), Fig.S1(c) to Fig.S1(h), or a *P*-value < 0.15 on univariate analysis in Fig.S1(b) were entered into multivariate logistical analysis by a simultaneous method.


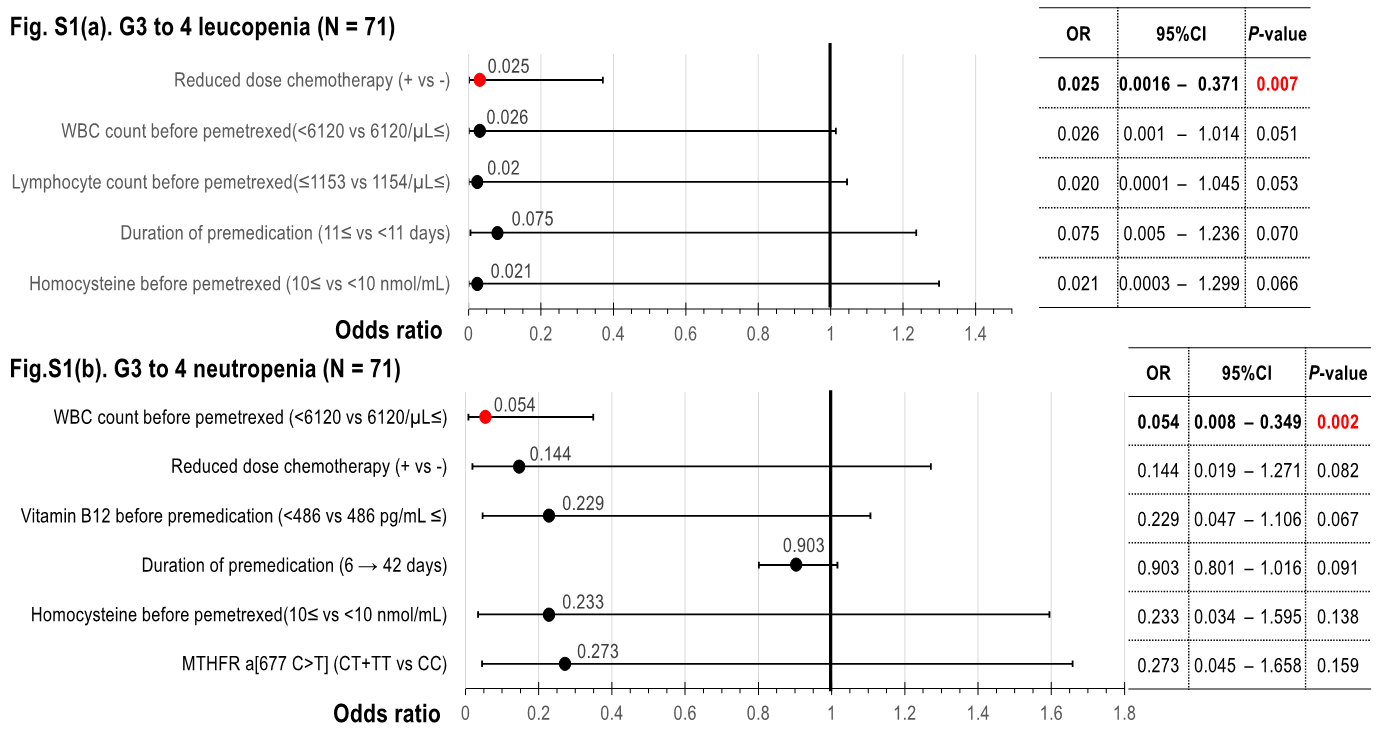


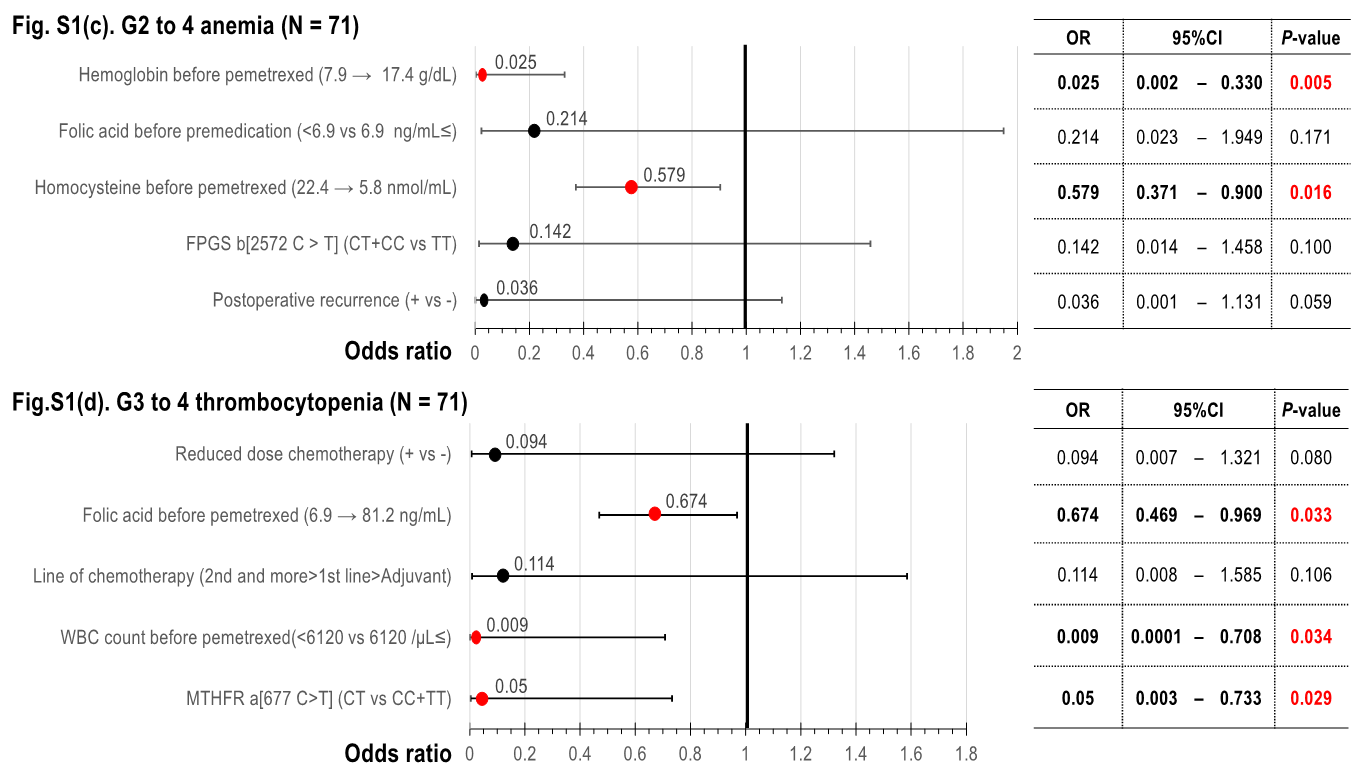


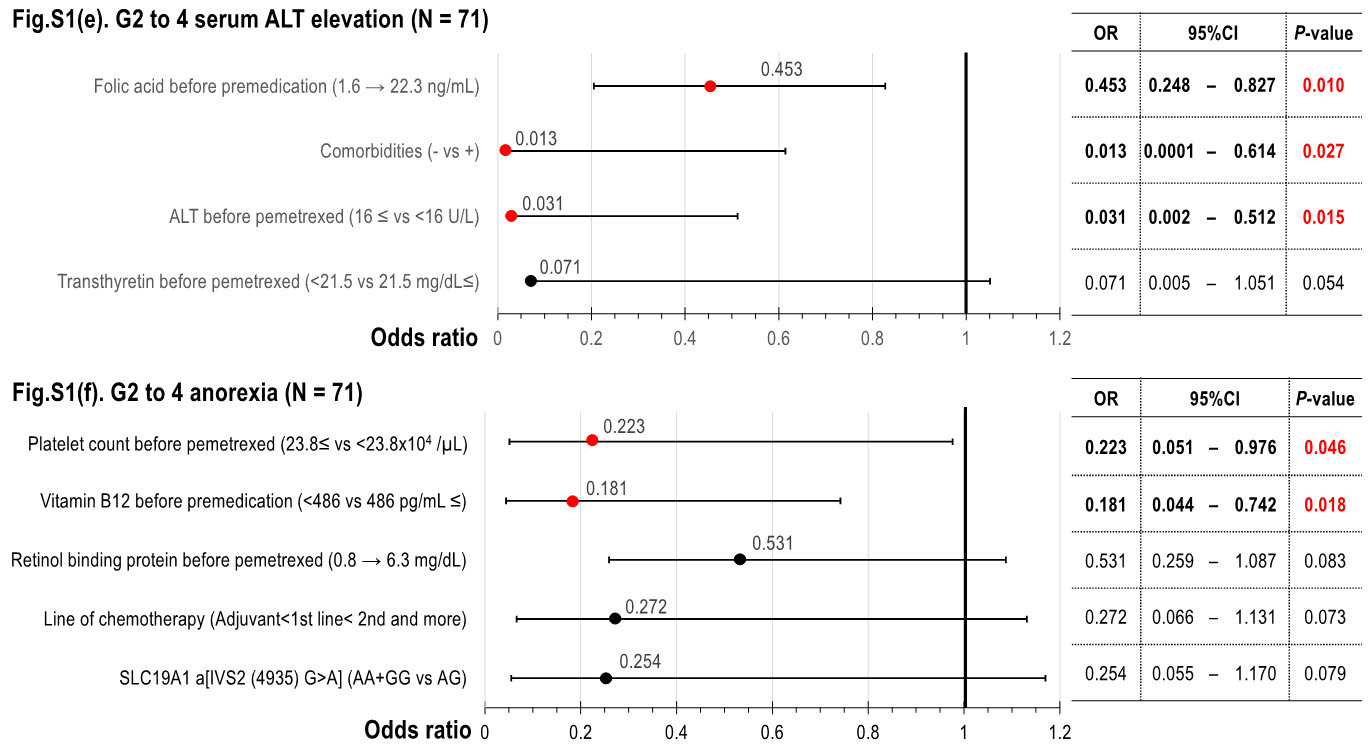


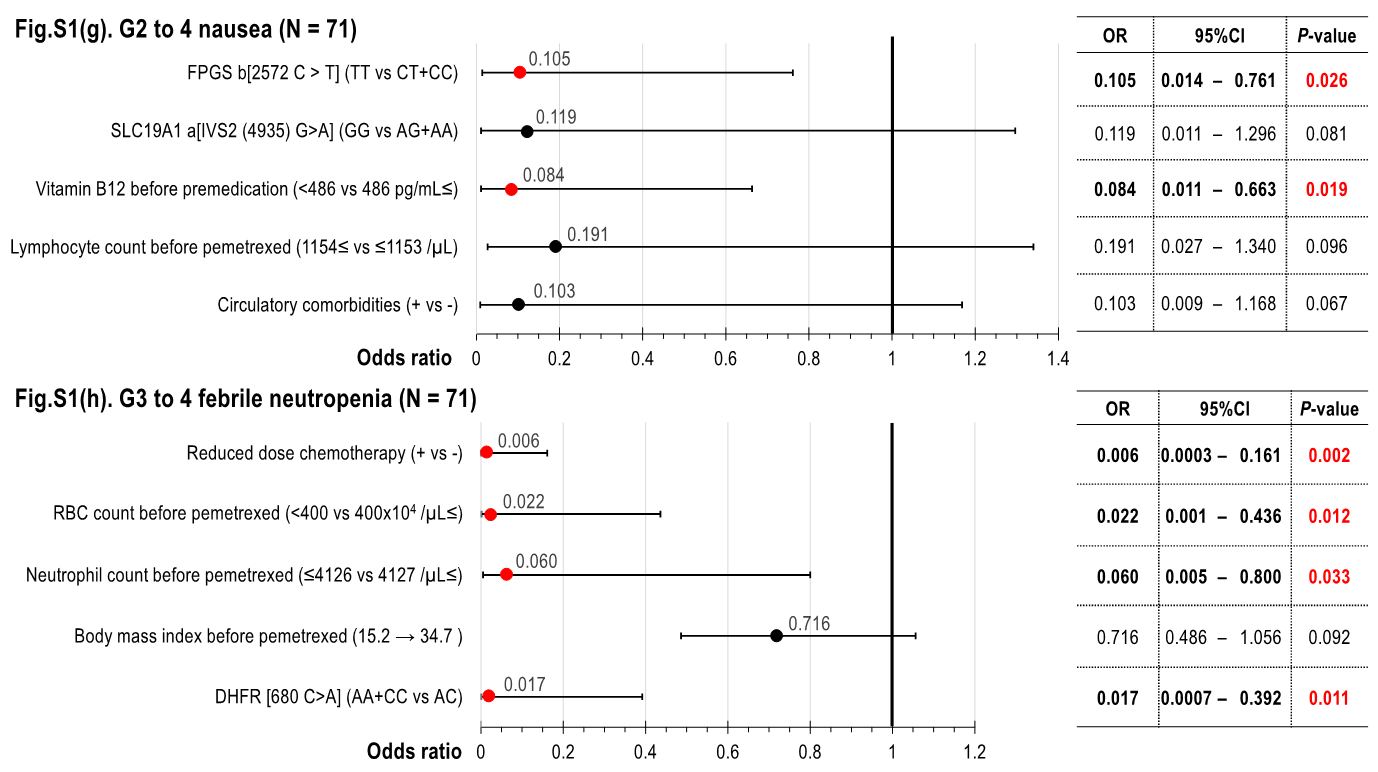

Supplement: Supplementary file 3 — Additional file 3: Supplement Fig S1. Propensity score-adjusted risk factors for toxicities at the 1st cycle by multivariate logistic regression analysis (N = 71). [file 12885_2023_11257_MOESM3_ESM.docx]
